# Apoptosis Triggers Specific, Rapid, and Global mRNA Decay with 3′ Uridylated Intermediates Degraded by DIS3L2

**DOI:** 10.1016/j.celrep.2015.04.026

**Published:** 2015-05-07

**Authors:** Marshall P. Thomas, Xing Liu, Jennifer Whangbo, Geoffrey McCrossan, Keri B. Sanborn, Emre Basar, Michael Walch, Judy Lieberman

**Affiliations:** 1 Program in Cellular and Molecular Medicine, Boston Children’s Hospital, Boston, MA 02115, USA

## Abstract

Apoptosis is a tightly coordinated cell death program that damages mitochondria, DNA, proteins, and membrane lipids. Little is known about the fate of RNA as cells die. Here, we show that mRNAs, but not noncoding RNAs, are rapidly and globally degraded during apoptosis. mRNA decay is triggered early in apoptosis, preceding membrane lipid scrambling, genomic DNA fragmentation, and apoptotic changes to translation initiation factors. mRNA decay depends on mitochondrial outer membrane permeabilization and is amplified by caspase activation. 3′ truncated mRNA decay intermediates with nontemplated uridylate-rich tails are generated during apoptosis. These tails are added by the terminal uridylyl transferases (TUTases) ZCCHC6 and ZCCHC11, and the uridylated transcript intermediates are degraded by the 3′ to 5′ exonuclease DIS3L2. Knockdown of *DIS3L2* or the TUTases inhibits apoptotic mRNA decay, translation arrest, and cell death, whereas *DIS3L2* overexpression enhances cell death. Our results suggest that global mRNA decay is an overlooked hallmark of apoptosis.

## Graphical abstract

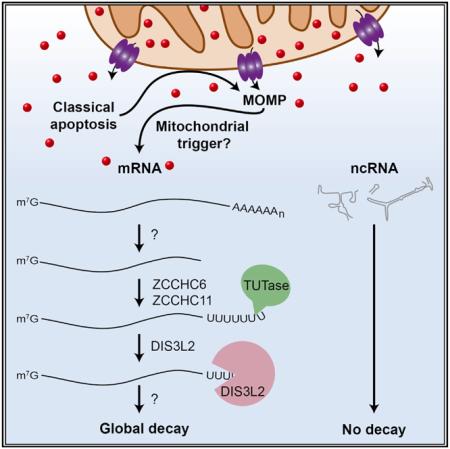

## INTRODUCTION

Mitochondrial outer membrane permeabilization (MOMP) and caspase activation are prominent shared events triggered by classical apoptotic stimuli, including DNA-damaging agents, death receptor signaling, and cytotoxic lymphocyte attack (Taylor et al., 2008). MOMP releases cytochrome *c* from the mitochondrial intermembrane space into the cytosol, where it drives the assembly of the apoptosome, the molecular scaffold that activates caspase 9, which cleaves and activates the effector caspase zymogens, notably caspase 3 (Riedl and Shi, 2004). The effector caspases cleave hundreds of substrates to cause cell death. The apoptotic program dismantles the cellular repair machinery as the cell self-destructs. Pre-mRNA splicing and RNA nuclear export are inhibited to prevent stress-responsive mRNAs from being translated (Rajani et al., 2012). New protein synthesis is blocked, ostensibly through translation initiation factor alterations that include eIF4G cleavage and eIF2α phosphorylation (Holcik and Sonenberg, 2005; Morley et al., 2005; Taylor et al., 2008). However, eiF4G cleavage is dispensable for translation arrest (Jeffrey et al., 2002), and eIF2α phosphorylation and eIF4G cleavage occur after translation is inhibited (Saelens et al., 2001). Thus, other mechanisms are needed to explain the block in translation during apoptosis (Thomas and Lieberman, 2013).

Human mRNAs are generally very stable, with a mean half-life of ~7 hr (Tani et al., 2012). Under normal conditions, most mRNAs decay via deadenylation followed by decapping and exonucleolytic decay from the 5′ and 3′ ends by XRN1 and the exosome, respectively (Schoenberg and Maquat, 2012). Little is known about what happens to RNA during apoptosis. 28S rRNA is cleaved late in cell death (Degen et al., 2000), but not in all dying cells. A few studies have suggested that the levels of some mRNAs decline during cell death (Bushell et al., 2004; Del Prete et al., 2002).

Recent work suggests that 3′ uridylation might also act as a signal for RNA turnover (Norbury, 2013). Nontemplated uridylate residues added by terminal uridylyl transferases (TUTases) have been found on histone mRNAs (Mullen and Marzluff, 2008; Rissland and Norbury, 2009; Schmidt et al., 2011; Slevin et al., 2014), pre-miRNAs (Thornton et al., 2012), and mRNAs at miRNA cleavage sites (Shen and Goodman, 2004). The TUTases ZCCHC11 (TUT4) and ZCCHC6 (TUT7) uridylate miRNAs (Thornton et al., 2012, 2014), whereas ZCCHC11 uridylates histone mRNAs (Schmidt et al., 2011). Human cells express three homologous 3′ to 5′ exoribonucleases: DIS3, DIS3L1, and DIS3L2. The first two are primarily associated with the nuclear (DIS3) and cytosolic (DIS3L1) exosome, but DIS3L2 is not (Lubas et al., 2013). DIS3L2, which preferentially degrades RNAs with 3′ uridylate residues, has been implicated in degradation of uridylated pre-miRNAs (Chang et al., 2013; Ustianenko et al., 2013) in human cells and mRNAs in fission yeast (Malecki et al., 2013). Knock-down of human *DIS3L2* also prolongs the half-life of mammalian polyadenylated mRNAs (Lubas et al., 2013), suggesting that it might also degrade mRNAs.

Here we show that global decay of mRNAs, but not noncoding RNAs (ncRNAs), occurs early after induction of apoptosis induced by diverse classical apoptotic stimuli. Decay is triggered by MOMP and begins around the time of caspase activation and before DNA degradation. mRNA decay intermediates are uridylated near the stop codon by the TUTases ZCCHC6 and ZCCHC11. The uridylated intermediates are further degraded by DIS3L2. mRNA decay promotes cell death, since cells better survive apoptotic stimuli after knockdown of *DIS3L2*, *ZCCHC6*, or *ZCCHC11*. Conversely, *DIS3L2* overexpression and transcription inhibitors enhance apoptosis. These results support the concept that global mRNA decay is a hallmark of cell death that may amplify apoptotic signaling. Further work is required to delineate the trigger and the complete apoptotic mRNA decay pathway.

## RESULTS

### Global mRNA Decay during Apoptosis

**Figure 1:**
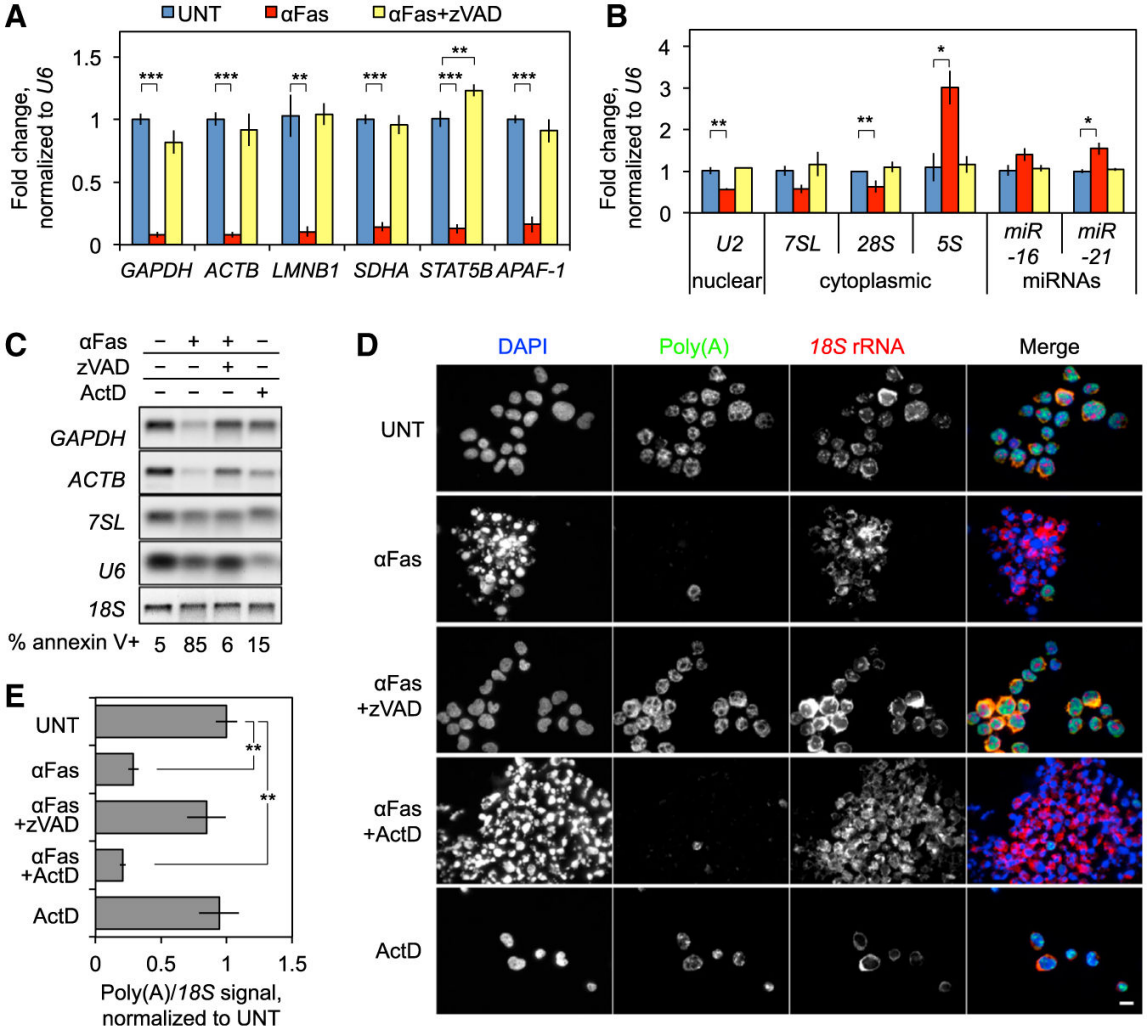
Global mRNA Decay in Apoptotic Jurkat T Cells (A–C) Jurkat cells were treated with αFas ± zVAD for 4 hr, total RNA was harvested, and mRNAs (A) and ncRNAs (B) were analyzed by qRT-PCR (UNT, untreated) and northern blot (C). RNA expression was normalized to *U6*; error bars represent the SEM of at least three independent experiments. In (C), *18S* rRNA was visualized by ethidium bromide staining and ActD-treated cells were included as a control for basal RNA stability. Cell death was confirmed by annexin V staining (C, bottom). mRNAs declined dramatically, whereas ncRNAs were more stable. (D and E) Poly(A) mRNA and *18S* rRNA were visualized by FISH in Jurkat cells treated with αFas ± zVAD for 3 hr; ActD was included as a control for RNA stability. Representative images (D) and quantification of the poly(A)/18S ratio averaged over multiple images from four independent experiments (E) are shown. Error bars represent SEM. Scale bar, 10 μm. *p < 0.05; **p < 0.01; ***p < 0.001, relative to untreated (UNT). See also Figure S1.

We first measured housekeeping mRNAs and ncRNAs by quantitative RT-PCR (qRT-PCR) and northern blot of total RNA in Jurkat cells treated with agonistic Fas antibody (αFas) for 4 hr with or without the pan-caspase inhibitor z-VAD-fmk (zVAD) (Figures 1A–1C). We found that 85% of αFas-treated cells stained with annexin V and death was completely rescued by zVAD (Figure 1C). qRT-PCR experiments were normalized to U6 small nuclear RNA (snRNA), which remained relatively stable during apoptosis. Six mRNAs, including *GAPDH* and *ACTB* (both with reported half-lives of >12 hr; Fan et al., 2002; Tani et al., 2012), declined by ~10-fold (Figure 1A). In contrast, some ncRNAs increased (*miR-21* and *5S* rRNA) and others (*U2* and *28S*) declined, but much less so than the mRNAs (Figure 1B). When the same cells were treated with Actinomycin D (ActD) for 4 hr to block transcription, mRNAs only declined slightly (Figure 1C), indicating that the mRNA half-life was drastically reduced. To test whether mRNA levels change globally during apoptosis, we used fluorescence in situ hybridization (FISH) to probe for *18S* rRNA and poly(A) mRNA (with a dT_50_ oligonucleotide) in Jurkat cells treated with αFas ± zVAD or ActD for 3 hr (Figures 1D, 1E, and S1A). Living cells had strong poly(A) and *18S* staining, whereas apoptotic cells retained rRNA staining but lost mRNA staining, which was rescued by zVAD. ActD had no significant effect on the relative strength of the poly(A) and *18S* signals. To determine whether the reduction in mRNA was caused by mRNA release into apoptotic blebs, we added αFas to Jurkat cells in the presence of blebbistatin, a myosin II inhibitor that blocks blebbing (Orlando et al., 2006). Blebbistatin did not inhibit the disappearance of mRNA (Figure S1B), suggesting that mRNA was not exported in blebs.

**Figure 2:**
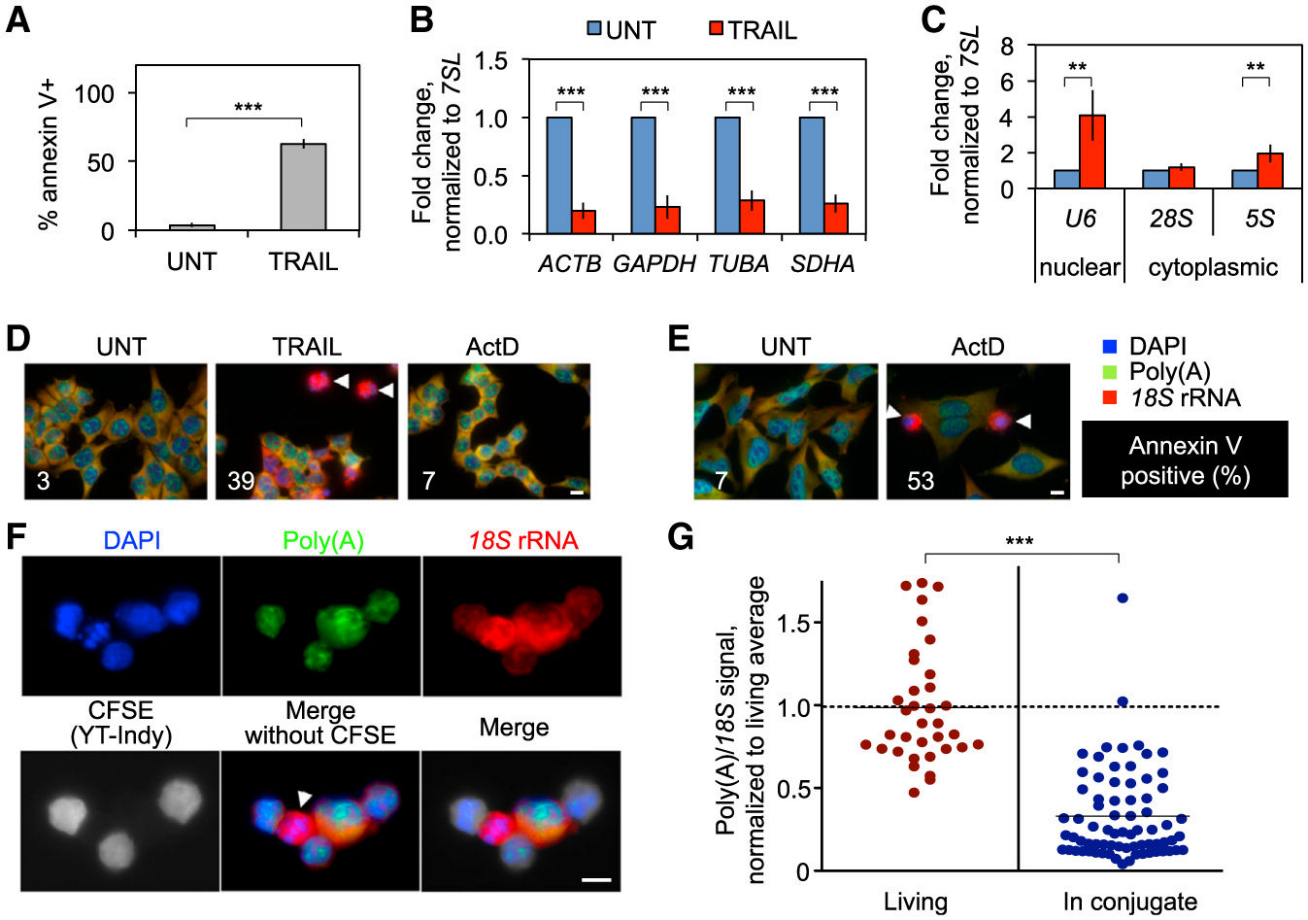
Global mRNA Decay Is a Shared Feature of Apoptosis in Diverse Cell Types (A–C) HCT116 cells were treated with TRAIL for 4 hr. Apoptosis was measured by annexin V staining (A), and mRNA (B) and ncRNA (C) levels were assayed by qRT-PCR. RNA expression was normalized to *7SL*. All mRNA levels declined, whereas ncRNAs increased or were constant. Error bars represent the SEM of at least three independent experiments. (D and E) HCT116 cells treated for 2.5 hr with TRAIL or a nontoxic concentration of ActD (D), and HeLa cells treated for 6 hr with a cytotoxic concentration of ActD (E) were analyzed by FISH. The percentage of annexin V+ cells is shown for each condition. Images are representative of at least three independent experiments. (F and G) CFSE-labeled YT-Indy cells were mixed with 721.221 cells for 3 hr and stained by FISH. Apoptosis of the target 721.221 cells was confirmed by ^51^Cr release (data not shown). Shown is a representative image (F) and quantification of the poly(A)/*18S* ratio in nonapoptotic 721.221 cells compared with target cells in conjugates with killer cells over three independent experiments (G). Poly(A) mRNA declined drastically, whereas rRNA was constant in apoptotic cells. In (D)–(F), apoptotic cells are indicated by arrowheads. Scale bar, 10 μm. **p < 0.01; ***p < 0.001, relative to untreated (UNT) (A–C) or Living (G). See also Figure S2.

To test whether global mRNA decay occurs in different cell types responding to diverse cytotoxic stimuli, we also treated HCT116 cells with a death receptor ligand (TRAIL) and two endoplasmic reticulum (ER) stressors (tunicamycin and thapsigargin), and measured apoptosis and RNA levels by annexin V staining and qRT-PCR, respectively (Figures 2A–2C and S2A–S2F). All treatments triggered rapid cell death and decay of mRNAs, but not ncRNAs. To test whether this decay was global, we performed FISH on HCT116 cells that were treated with TRAIL or ActD for 2.5 hr, and on HeLa cells treated with a cytotoxic concentration of ActD for 6 hr (Figures 2D and 2E). In both cases, apoptotic cells lost poly(A) mRNA signal but retained strong rRNA staining. Cells without apoptotic nuclear morphology in the same fields retained a strong poly(A) signal, even after complete transcription inhibition with ActD. Global mRNA decay also occurred in cells undergoing cytotoxic attack. Carboxyfluorescein succinimidyl ester (CFSE)-labeled YT-Indy natural killer cells were incubated with 721.221 B cells for 3 hr at a ratio of 3:1 and stained by FISH (Figures 2F and 2G). At that time, 60% of 721.221 cells were apoptotic as assessed by ^51^Cr release (data not shown). Apoptotic 721.221 cells (identified by their condensed nuclei), but not living target cells, lost mRNA signal. The ratio of poly(A) signal to *18S* signal declined significantly in 721.221 cells when they were coincubated with YT-Indy cells (Figure 2G).

To determine whether the decay was specific for translated RNAs, we examined the effect of staurosporine (STS) on GFP reporter RNAs driven by Pol I, II, or III promoters in HeLa cells. The Pol II-driven reporters were terminated by a poly(A) sequence (Pol II GFP), a self-cleaving hammerhead ribozyme (HR), or an HR preceded by 60 adenylates (HR-A_60_) or uridylates (HR-U_60_) (Figures S2G–S2J; Gaglia et al., 2012; Lee and Glaunsinger, 2009). Although all of the reporters were transcribed (Figure S2H), only Pol II GFP, HR-A_60_, and HR-U_60_ were translated (Figures S2I and S2J). Only the translated mRNAs declined in apoptotic cells (Figure S2H). Collectively, these results show that mRNAs, but not ncRNAs, are degraded during apoptosis in multiple cell types (Jurkat, HCT116, 721.221 B, and HeLa cells) responding to different apoptotic stimuli.

### mRNA Decay Occurs Early in Apoptosis and Requires MOMP

**Figure 3:**
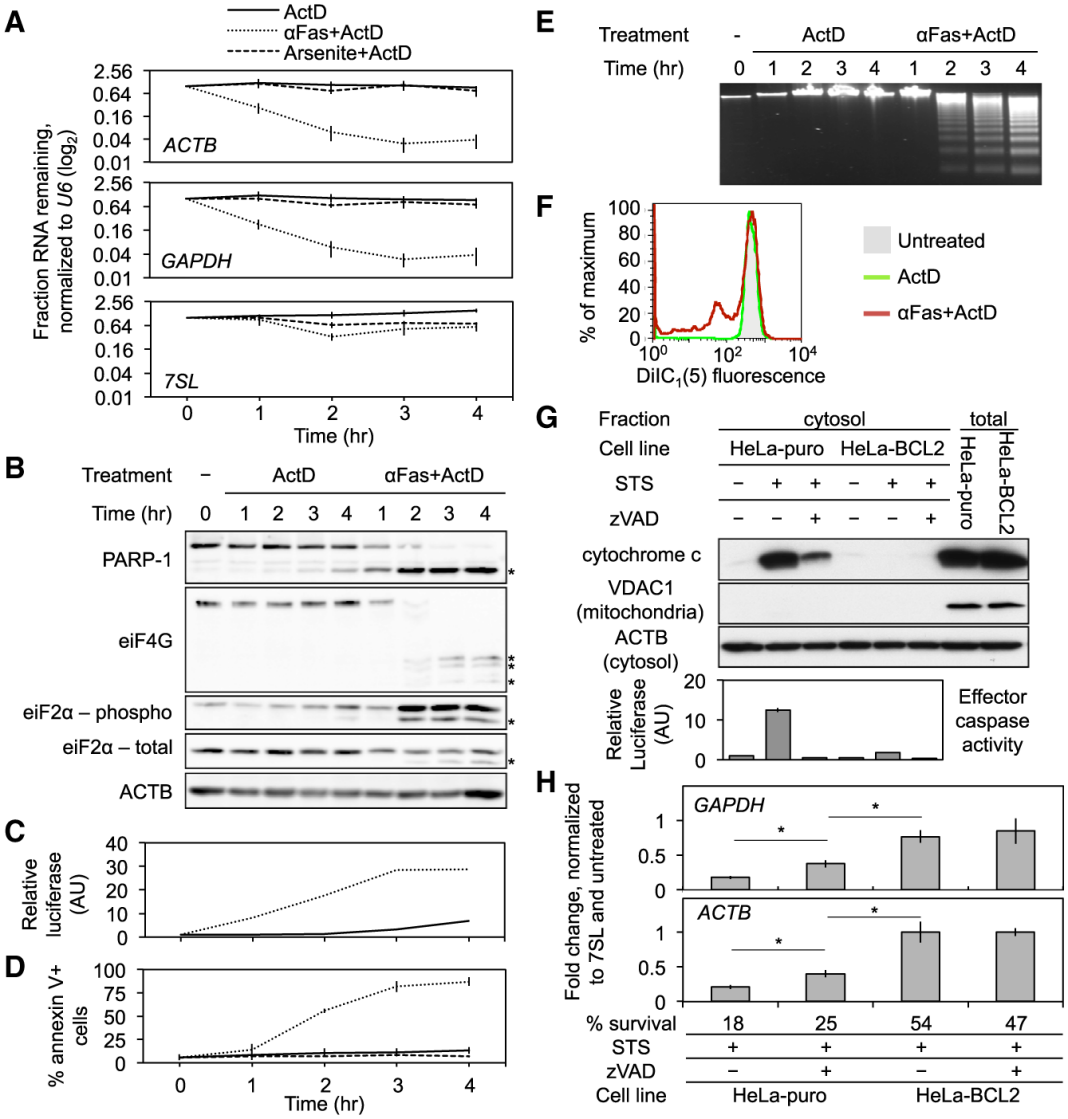
mRNA Decay Begins Early in Cell Death and Requires MOMP (A–E) Jurkat cells were treated with ActD ± αFas or arsenite for 4 hr and harvested hourly for qRT-PCR (A), immunoblot (B), caspase activity by luminescent assay (AU, arbitrary units) (C), annexin V staining by flow cytometry (D), and DNA fragmentation by electrophoresis (E). Asterisks in (B) indicate caspase cleavage products. mRNA decay was concurrent with caspase activation, but preceded DNA cleavage and phosphatidylserine externalization. Error bars represent the SEM of at least three independent experiments. Results in (B) and (E) are representative of multiple experiments, and the experiment shown in (C) was performed once. (F) Jurkat cells were treated for 1 hr with ActD ± αFas and stained with DiIC_1_(5) to measure mitochondrial depolarization. The transmembrane potential began dissipating within 1 hr after αFas treatment. Results are representative of at least three independent experiments. (G and H) HeLa-BCL2 and HeLa-puro were treated for 6 hr with STS ± zVAD. (G) Fractionated cells were analyzed by immunoblot (top) for cytochrome *c* release. Effector caspase activity was measured by luminescent reporter assay (bottom). Error bars represent the SEM of separate wells from one experiment. (H) RNAs were assayed by qRT-PCR. Error bars represent the SEM of at least three independent experiments (*p < 0.05). Results were normalized to untreated cells. *BCL2* overexpression inhibited mRNA decay to a greater extent than did zVAD. Survival (assayed by CytoTox-Glo assay performed after 6 hr of STS treatment followed by a 24 hr recovery period) was normalized to untreated cells. See also Figure S3.

We next determined the kinetics of the mRNA decline relative to hallmarks of apoptosis. Jurkat cells treated with ActD ± αFas were harvested hourly over 4 hr and analyzed for housekeeping gene mRNA stability by qRT-PCR, cleavage of PARP-1 and eiF4G by immunoblot, phosphorylation of eiF2α by immunoblot, caspase activation by a luminescent assay, annexin V binding, and DNA fragmentation (Figures 3A–3E). *ACTB* and *GAPDH* mRNAs began to decline within 1 hr after addition of αFas and leveled off by 3 hr, whereas *7SL* ncRNA was stable (Figure 3A). Nonapoptotic oxidative stress caused by arsenite (+ActD) and caspase-independent programmed cell death induced by the killer protease granzyme A did not affect mRNA levels (Figure 3A and data not shown). Caspase activation and PARP-1 cleavage began at 1 hr and were complete by 3 hr (Figures 3B and 3C). eIF2α phosphorylation and eIF4G cleavage were first detected after 2 hr (Figure 3B). Similarly, phosphatidylserine externalization and DNA fragmentation were not detected until 2 hr after αFas was added (Figures 3D and 3E). Mitochondrial depolarization began within 1 hr after αFas treatment (Figure 3F). Thus, mRNA decay occurred early in cell death coincidently with caspase activation and mitochondrial disruption.

Because MOMP is an early step in apoptosis (Aldridge et al., 2011), we asked whether MOMP is required for mRNA decay. Stressors such as STS activate cell death by initiating MOMP upstream of caspase activity, whereas αFas activates caspase 8 to trigger MOMP. Thus, zVAD blocks MOMP induced by αFas, but not by STS (Arnoult et al., 2003; Holler et al., 2000). We compared HeLa cells stably expressing *BCL2* (HeLa-BCL2) or an empty vector (HeLa-puro). In agreement with previous studies (Arnoult et al., 2003), *BCL2* overexpression blocked the release of cytochrome *c* and effector caspase activation caused by STS (Figure 3G). In contrast, zVAD blocked STS-triggered caspase activation, but did not block cytochrome *c* release or cell death. mRNA decay was rescued by *BCL2* expression, but only partially by zVAD (Figure 3H). Knockdown of *BAX* and *BAK* also rescued mRNA decay to a greater extent than did zVAD (Figure S3). Thus, mRNA decay occurs early in apoptosis and depends on MOMP.

### Depletion of Cellular mRNA Promotes Apoptosis

**Figure 4:**
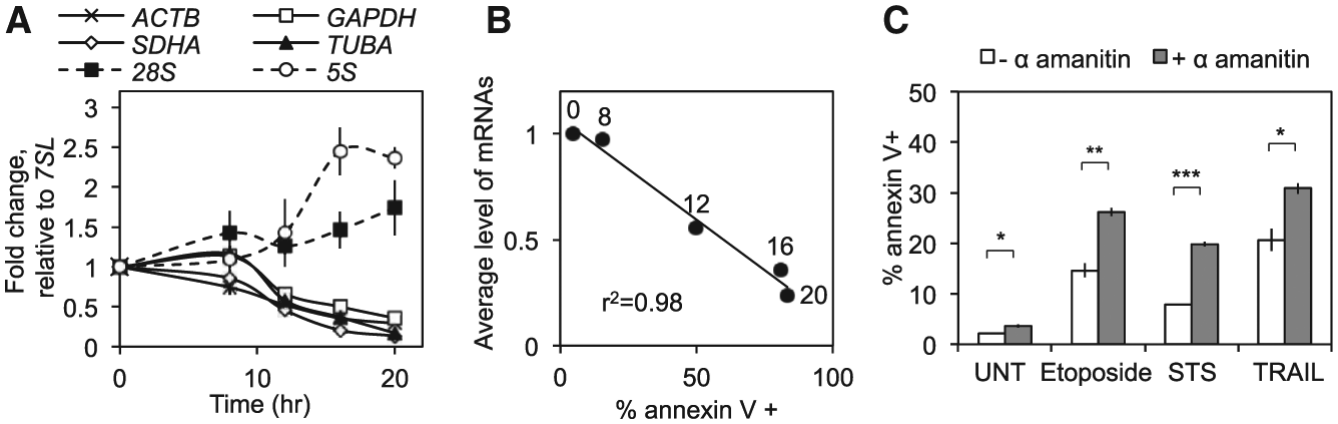
Cell Viability Correlates with mRNA Levels (A and B) HeLa cells were treated with the transcriptional inhibitor α-amanitin for 20 hr and harvested at regular intervals, and RNA levels were measured by qRT-PCR (A). Cell death was measured by annexin V staining and flow cytometry, and then plotted relative to the average level of the four mRNAs analyzed (B). Labels on data points represent the time of α-amanitin treatment in hours. Cell death increased as mRNA levels dropped. Results are mean ± SEM from triplicate experiments. (C) HeLa cells were treated with or without α-amanitin and STS, TRAIL, etoposide, or nothing (UNT) for 8 hr. Cell death was measured by annexin V staining and flow cytometry. In all cases, transcription inhibition significantly enhanced the progression to apoptosis. Error bars represent the SEM of at least three independent experiments. *p < 0.05; **p < 0.01; ***p < 0.001.

We hypothesized that the global mRNA depletion we observed would contribute to apoptosis. To test the importance of maintaining the pool of mRNAs for cell viability, we treated HeLa cells with the Pol II inhibitor α-amanitin and examined how changes in RNA levels over time (assessed by qRT-PCR; Figure 4A) correlated with cell death (assessed by annexin V staining and flow cytometry; Figure 4B). The decline in mRNA levels triggered by transcription inhibition correlated closely with (r^2^ = 0.98; Figure 4B). We also found that transcription inhibition significantly enhanced the cytotoxic effect of STS, TRAIL, and etoposide (Figure 4C). Thus, a global loss of mRNA is incompatible with continued cell survival.

### mRNA Decay Intermediates Contain Nontemplated 3′ Uridylates

**Figure 5:**
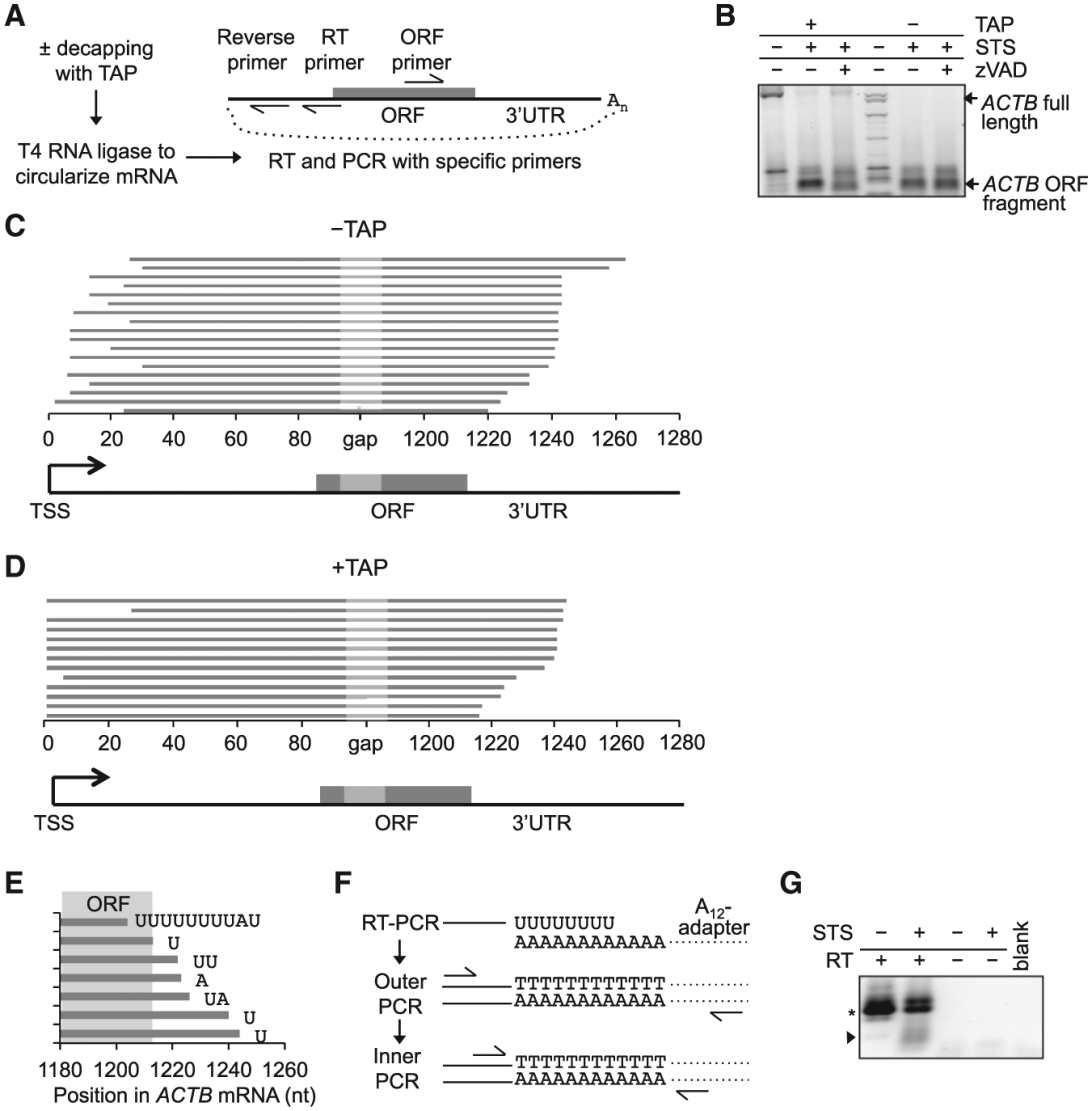
3′ Uridylation of Apoptotic Decay Intermediates (A) Schematic of the cRACE method used to identify *ACTB* mRNA decay intermediates. cRACE was performed on RNA from HeLa cells treated for 6 hr with STS ± zVAD. (B–E) *ACTB* decay products were amplified using an ORF-targeted primer and a reverse primer in the 5′ UTR, and analyzed by gel electrophoresis (B). The *ACTB* fragment was gel purified, cloned, and sequenced from reactions that were treated (D) or not (C) with TAP prior to cRACE. Line diagrams (C and D) depict all ORF-primer amplified cRACE clones that mapped to the *ACTB* transcript, excluding clones with nontemplated 3′ extensions. The decay intermediates were truncated just 3′ of the stop codon. Decapped decay intermediates (−TAP) had shortened 5′ ends, whereas most clones derived from TAP-treated RNA had a complete 5′ sequence. (E) Some apoptotic *ACTB* mRNA fragments had nontemplated 3′ tails. Shown are nontemplated RNAs obtained from TAP-treated RNA. Templated residues matching the *ACTB* sequence are depicted as a line, and uridylate-rich nontemplated tails are indicated. All clones in (C)–(E) were derived from one experiment. (F and G) Amplification of poly(U)-tailed RNAs using an A_12_-adaptor reverse RT primer. (F) Schematic of the RT-PCR method. After RT, nested PCR was used to amplify uridylated decay products, with forward primers targeting the *ACTB* ORF and reverse primers targeting the adaptor sequence. (G) Gel electrophoresis of RT-PCR products. Arrow indicates new uridylated *ACTB* mRNA decay fragments in apoptotic cells. The asterisk denotes products primed from oligouridylate tracts elsewhere in the *ACTB* 3′UTR. Results are representative of at least three independent experiments. See also Figure S4.

To begin to understand the mechanism of mRNA decay during cell death, we searched for decay intermediates using circular rapid amplification of cDNA ends (cRACE) (Rissland and Norbury, 2009) to ligate and amplify the 5′ and 3′ ends of *ACTB* mRNA (Figure 5A). cRACE was performed with and without tobacco acid pyrophosphatase (TAP) pre-treatment to distinguish between capped and decapped intermediates (only decapped mRNAs can be ligated without TAP) in HeLa cells treated with STS ± zVAD for 6 hr. Using a forward primer directed to the *ACTB* 3′ UTR near its polyadenylation site, we amplified residual full-length *ACTB* mRNAs from both untreated and STS-treated apoptotic cells, but only using TAP-treated RNA (Figures S4A and S4B). There were no visible decapped decay intermediates. The cloned and sequenced cRACE products contained poly(A) tails of similar lengths independently of the treatment used (Figure S4C). However, when RNA was amplified with a PCR primer located near the stop codon in the *ACTB* open reading frame (ORF), we detected decay intermediates in apoptotic cell RNA even in cells treated with zVAD (Figure 5B). These intermediates were also amplified without TAP pre-treatment, indicating that at least some of the decay intermediates were decapped. When the cRACE products from STS-treated cells were cloned, the junctions that mapped exactly to the *ACTB* mRNA had two notable features (Figures 5C and 5D). First, their 3′ termini were within 50 nt of the *ACTB* stop codon, suggesting stalled decay near the ORF. Second, most junctions captured from TAP-treated RNA had 5′ termini that mapped exactly to the *ACTB* transcription start site (TSS), whereas all of the 5′ termini captured from TAP-untreated RNA began after the TSS. These results suggest that decay proceeds from 3′ to 5′ on deadenylated mRNAs before decapping occurs. Surprisingly, some clones had nontemplated residues between the 3′ and 5′ termini. These added bases were rich in uridylates (Figure 5E). Similar results were obtained by cRACE and sequencing of the decay intermediates of another mRNA (*EEF1A*; Figures S4D–S4G). Thus, nontemplated 3′ uridylates are added in the region near the stop codon during apoptotic mRNA decay. To confirm that 3′ uridylated *ACTB* decay intermediates were produced during apoptosis, we performed RT-PCR on RNA isolated from HeLa cells treated or not with STS using an A_12_-adaptor for RT to prime poly(U) sequences, and nested primers targeting the *ACTB* ORF and the adaptor primer (Figure 5F). Novel decay products reproducibly appeared only in STS-treated cells (Figure 5G). Cloning and sequencing confirmed that these were similar to the cRACE products (data not shown).

### ZCCHC6 and ZCCHC11 Contribute to mRNA Decay and Apoptosis

**Figure 6:**
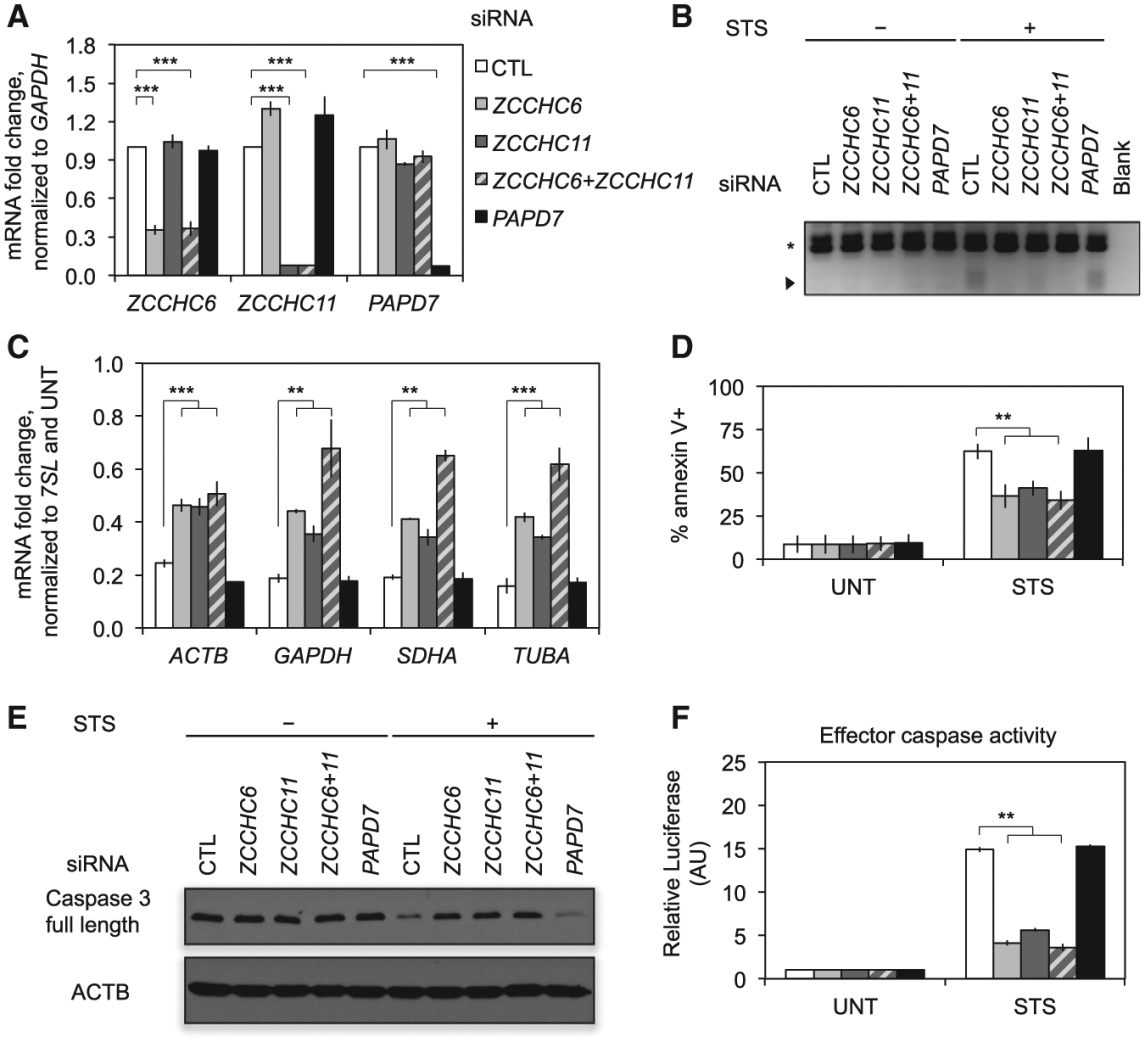
*ZCCHC6/ZCCHC11* Knockdown Inhibits Cell Death and mRNA Decay (A) HeLa cells were transfected with control (CTL), *ZCCHC6*, and/or *ZCCHC11* or *PAPD7* siRNAs and harvested 72 hr later. RNA was then analyzed by qRT-PCR to assess knockdown. (B) Accumulation of uridylated decay intermediates was assessed by RT-PCR with an A_12_-adaptor primer (as in Figure 5F). Uridylated intermediates that arose after STS treatment were reduced after knockdown of *ZCCHC6* and/or *ZCCHC11*. The arrowhead denotes new products and the asterisk denotes products primed from oligouridylate tracts in the *ACTB* 3′ UTR. Results are representative of three independent experiments. (C–F) After knockdown and STS treatment, mRNA levels were assayed by qRT-PCR (C). Cells were analyzed for cell death by annexin V staining and flow cytometry (D). Caspase activation was assessed by immunoblot for caspase 3 (E) and a luminescent caspase activity assay (F). *ZCCHC6* and/or *ZCCHC11* knockdown partially restored mRNA levels, rescued cell death, and reduced caspase 3 cleavage and activation. Error bars represent the SEM of at least three independent experiments. **p < 0.01; ***p < 0.001, relative to CTL knockdown.

We hypothesized that one or more TUTases add these non-templated uridylate-rich tails. We focused on ZCCHC6 and ZCCHC11 because they uridylate other cytosolic RNAs (Schmidt et al., 2011; Thornton et al., 2012, 2014). We transfected HeLa cells with a control siRNA or siRNAs targeting *ZCCHC6* and/or *ZCCHC11*, or the TUTase *PAPD7* (Figure 6A). Knockdown of *ZCCHC6*, *ZCCHC11*, or both reduced apoptotic 3′ uridylation of the *ACTB* mRNA (Figure 6B) and partially rescued mRNA levels after STS treatment (Figure 6C), whereas *PAPD7* knock-down had no effect. *ZCCHC6* and *ZCCHC11* siRNAs, alone and in combination, reduced annexin V staining (Figure 6D) and caspase 3 cleavage and activation (Figures 6E and 6F) in response to STS. Again, *PAPD7* siRNAs had no effect. Collectively, these results suggest that ZCCHC6 and ZCCHC11 collaborate to uridylate mRNAs during cell death, promoting mRNA decay and apoptosis.

### *DIS3L2* Knockdown Inhibits mRNA Decay and Cell Death

**Figure 7:**
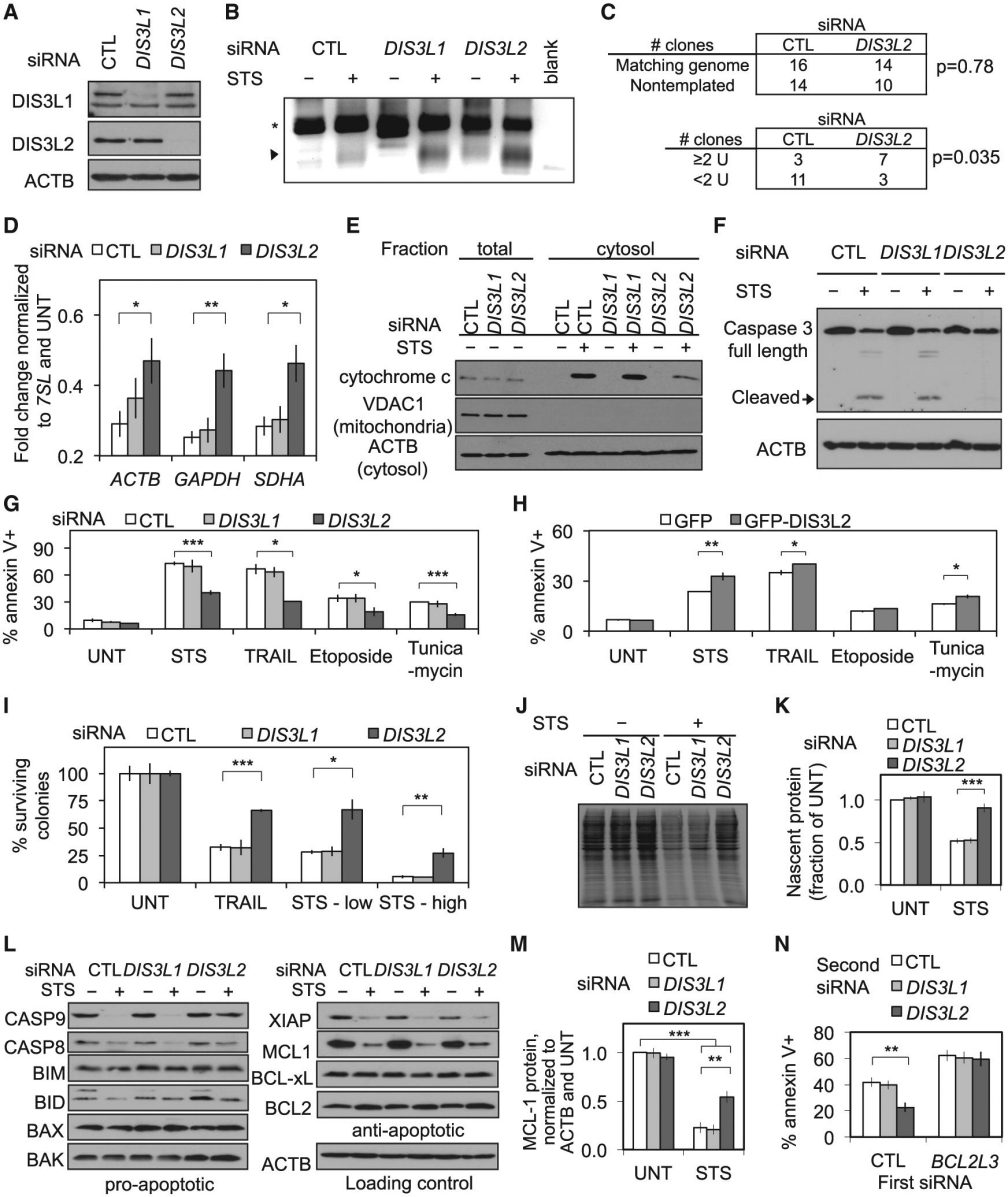
*DIS3L2* Knockdown in HeLa Cells Inhibits Cell Death and mRNA Decay (A) HeLa cells transfected with control (CTL), *DIS3L1*, or *DIS3L2* siRNAs were harvested 72 hr later for immunoblot, confirming robust knockdown of DIS3L1 and DIS3L2 proteins. (B) Accumulation of uridylated decay intermediates was assessed by RT-PCR with an A_12_-adaptor primer (as in Figure 5F). Uridylated intermediates accumulated in STS-treated cells regardless of the siRNA transfected, but accumulated to a greater extent after *DIS3L2* knockdown. The arrowhead denotes new products and the asterisk denotes products primed from oligouridylate tracts in the *ACTB* 3′ UTR. Results are representative of at least three independent experiments. (C) Increased uridylation was validated by cloning cRACE products (as in Figure 5). For knockdown in this experiment and Figures S5A–S5G, we used an independent set of siRNAs to verify that the results were not due to off-target effects of gene knockdown. Products with two or more nontemplated uridylates were more abundant in STS-treated cells after *DIS3L2* knockdown, but the proportion of products with any nontemplated residues on the 3′ end was similar between the CTL and *DIS3L2* knockdown samples. (D–F) After knockdown and STS treatment, mRNA levels were assayed by qRT-PCR (D). Cytochrome *c* release was measured by cellular fractionation followed by immunoblot (E) and caspase 3 cleavage was measured by immunoblot (F). β-Actin (ACTB) was probed as a loading control and VDAC1 was probed to verify fractionation. *DIS3L2* knockdown partially restored mRNA levels and reduced cytochrome *c* release and caspase 3 cleavage. (G) At 72 hr after transfection with the indicated siRNAs, cells were incubated with STS or TRAIL for 3 hr or etoposide or tunicamycin for 8 hr. Cells were analyzed by annexin V staining and flow cytometry. *DIS3L2* knockdown significantly reduced phosphatidylserine externalization and cell survival in response to all treatments. (H) Cells were transfected with a plasmid expressing GFP or GFP-tagged DIS3L2 and treated 72 hr later, as in (G). Cells were analyzed by annexin V staining and flow cytometry. Ectopic DIS3L2 expression significantly increased phosphatidylserine externalization in response to STS, TRAIL, and tunicamycin. (I) Cells were replated at low density 48 hr after knockdown and were treated 24 hr later with TRAIL or two doses of STS for 3 hr. After the drugs were removed, the cells were cultured and surviving colonies were counted. *DIS3L2* knockdown significantly enhanced clonogenic survival in response to all treatments. UNT, untreated. (J and K) siRNA-transfected cells were treated 2 days later with STS for 4 hr and protein incorporation was measured by ^35^S incorporation followed by PAGE and autoradiography. A representative experiment (J) and the results of three independent experiments (K) are shown. Significance relative to CTL siRNA is shown. (L) siRNA-transfected cells were treated 3 days later with STS for 4 hr and proteins were analyzed by immunoblot. (M) MCL-1 protein levels declined significantly after 4 hr of STS treatment. The decline was partially rescued by *DIS3L2*, but not *DIS3L1*, knockdown. (N) siRNA-transfected cells were treated 3 days later with STS for 3 hr and cell death was measured by annexin V staining and flow cytometry. *BCL2L3* knockdown abolished the protective effect of *DIS3L2* knockdown. Error bars represent the SEM of at least three independent experiments. *p < 0.05; **p < 0.01; ***p < 0.001. See also Figure S5.

Because DIS3L2 targets 3′ uridylated pre-miRNAs (Chang et al., 2013; Ustianenko et al., 2013), we hypothesized that DIS3L2 digests the uridylated mRNA decay products we observed in cell death. We knocked down *DIS3L1* (a ribonuclease in the cytosolic exosome, as control) and *DIS3L2* in HeLa cells (Figure 7A) and used RT-PCR with an A_12_-adaptor to amplify 3′ uridylated *ACTB* mRNA intermediates (Figure 7B). Novel uridylated *ACTB* products were detected in nonapoptotic cells transfected with *DIS3L2* siRNA, but not in those transfected with *DIS3L1* or control siRNA. The appearance of uridylated mRNA decay intermediates in living cells after *DIS3L2* knockdown suggests that DIS3L2 plays a role in basal mRNA decay. After STS treatment, the uridylated ACTB mRNA intermediates greatly increased with *DIS3L2* knockdown, and increased somewhat with *DIS3L1* knockdown. We confirmed the increase in uridylated *ACTB* mRNA intermediates with *DIS3L2* knockdown by cloning and sequencing cRACE products (Figures 7C and S5A–S5G). The supplemental data in Figures S5A–S5G were obtained using another *DIS3L2* siRNA to verify that the effect of knocking down DIS3L2 was not due to off-target effects. Knockdown of *DIS3L2*, but not *DIS3L1*, also significantly reduced STS-mediated mRNA decay of three housekeeping genes (*ACTB, GAPDH*, and *SDHA*; Figures 7D and S5E). Knockdown of *DIS3L2*, but not *DIS3L1*, increased the mRNA half-life in STS-treated HeLa cells (Figures S5H and S5I). To verify that the role of DIS3L2 in mRNA decay was not limited to STS-treated HeLa cells, we also examined *DIS3L1* and *DIS3L2* knockdown in HCT116 cells treated with TRAIL (Figures S5J–S5N). *DIS3L2* knockdown specifically reduced TRAIL-mediated decay of four housekeeping genes, but knockdown of *DIS3L1* only affected one of them (and to a lesser extent; Figure S5K). Thus, DIS3L2 is a mediator of mRNA decay in apoptosis and may also play a role in basal mRNA decay.

Next, we examined whether mRNA decay contributes to apoptosis. We compared cytochrome *c* release, caspase activation, annexin V staining, and clonogenic survival of HeLa or HCT116 cells transfected with control, *DIS3L1*, or *DIS3L2* siRNAs and then treated with a variety of apoptotic stimuli. STS-induced cytochrome *c* release in HeLa was reduced when *DIS3L2*, but not *DIS3L1*, was knocked down (Figure 7E). Caspase 3 cleavage was detected 4 hr after STS treatment in cells treated with *DIS3L1* or control siRNA, but was rescued by DIS3L2 knockdown (Figure 7F). DIS3L2 knockdown also reduced annexin V staining in HeLa cells after exposure to STS, etoposide, tunicamycin, and several doses of TRAIL (Figures 7G and S5O). Knockdown of *DIS3L1* or *DIS3*, the homologous exonuclease in the nuclear exosome (data not shown), did not affect annexin V staining. Conversely, *DIS3L2* overexpression in HeLa significantly increased annexin V staining in response to STS, TRAIL, and tunicamycin (Figure 7H). *DIS3L2* knockdown also specifically and significantly enhanced the clonogenic survival of HeLa cells after TRAIL or STS treatment (Figure 7I). *DIS3L2* knockdown reduced caspase activation in HeLa cells transfected with a different siRNA (Figures S5F and S5G), and caspase activation and cell death in HCT116 cells treated with TRAIL (Figures S5L–S5N). Taken together, these results show that mRNA decay, mediated by DIS3L2, strongly promotes apoptosis.

### DIS3L2-Mediated mRNA Decay Inhibits De Novo Protein Synthesis during Apoptosis

It is well known that translation is arrested during apoptosis. This has been attributed to post-translational modifications of translation initiation factors. However, global mRNA decay could also have a profound effect on translation. To explore how mRNA decay and its inhibition by *DIS3L2* knockdown affect translation during apoptosis, we measured global translation by incorporation of ^35^S-methionine and ^35^S-cysteine into HeLa cells after control, *DIS3L1*, or *DIS3L2* knockdown, with and without STS treatment (Figures 7J and 7K). In the absence of STS, knock-down of *DIS3L1* or *DIS3L2* did not affect translation. Following STS treatment, translation declined by about 50% in control cells and cells knocked down for *DIS3L1*. However, *DIS3L2* knockdown restored translation almost to the level observed in nonaptoptotic cells. Thus, global mRNA decay during apoptosis strongly inhibits new protein synthesis, and inhibition of translation depends on DIS3L2.

### *DIS3L2* Knockdown Rescues Cell Death through MCL-1

Inhibiting mRNA decay by knocking down *DIS3L2* inhibited cytochrome *c* release and caspase activation. To begin to understand the mechanism behind this, we examined the effect of *DIS3L1* and *DIS3L2* knockdown on pro- and antiapoptotic protein expression at baseline or after STS treatment (Figure 7L). Although there was no discernible change in most of the proteins examined, some of the proapoptotic proteins increased a little after STS treatment in cells depleted of DIS3L2, including caspases 8 and 9 and BAK. However, such changes would be expected to enhance, rather than reduce, apoptosis. Among the antiapoptotic proteins examined, XIAP, BCL-X_L_, and BCL2 did not change with *DIS3L2* knockdown, but MCL-1 decreased less after STS in *DIS3L2* knockdown cells compared with controls (Figures 7L and 7M). MCL-1, which inhibits MOMP, is degraded by the proteasome and caspases during apoptosis (Nijhawan et al., 2003; Weng et al., 2005). Indeed, MCL-1 levels declined substantially after STS in all cells. Degradation of its mRNA and consequent inhibition of translation would be expected to reduce MCL-1 expression, which could promote MOMP and downstream amplification of apoptosis. To evaluate the interactions between *DIS3L2* and *BCL2L3* (the gene encoding MCL-1) in apoptosis, we examined the effect on STS-mediated death of knocking down BCL2L3 in HeLa cells knocked down for *DIS3L1* or *DIS3L2* (Figure 7N). Rescue from death by *DIS3L2* knockdown was completely abrogated in cells that were also knocked down for *BCL2L3*. This result suggests that DIS3L2-mediated degradation of *BCL2L3* mRNA contributes to cell death in this setting.

## DISCUSSION

Here, we have shown that early, global mRNA decay occurs during classical apoptotic cell death. Global mRNA degradation, which did not apply to ncRNA, was instigated by a variety of proapoptotic signals (death receptor ligation, STS, etoposide, tunicamycin, thapsigargin, high-dose ActD, and cytotoxic attack) in several different cell types. It was specific to classical apoptosis and did not occur during nonapoptotic oxidative stress or caspase-independent programmed cell death triggered by granzyme A. We are unaware of other physiological settings in which mRNA is degraded so rapidly and globally. Furthermore, global mRNA decay contributes to translation arrest in apoptosis. Decay was inhibited by blocking MOMP with *BCL2* overexpression or *BAX/BAK* knockdown, but only partially attenuated by caspase inhibition. These findings suggest that a mitochondrial product released during MOMP initiates global mRNA decay. Future studies will need to define the trigger for mRNA decay.

In this study we identified an mRNA decay pathway that is activated during apoptosis. *ACTB* and *EEF1A* mRNA decay intermediates had nontemplated oligouridylated 3′ ends near the stop codon. Knockdown of *ZCCHC6* and *ZCCHC11* TUTases reduced apoptotic 3′ uridylation of mRNA decay products, mRNA decay, and cell death. Uridylated decay intermediates increased after *DIS3L2* knockdown, which also inhibited global mRNA decay and translation arrest. These results suggest that the TUTases uridylate decay intermediates near the stop codon; these are then digested by the DIS3L2 exoribonuclease, which has specificity for uridylated 3′ ends. mRNA decay can occur on translating polyribosomes (Hu et al., 2009, 2010; Lubas et al., 2013; Slevin et al., 2014). Uridylation of stalled transcripts and DIS3L2 digestion of uridylated mRNAs may serve to reinitiate degradation caused by stalling at ribosomes.

The TUTases and DIS3L2 are likely responsible for only some of the steps of apoptotic mRNA decay. We did not find that poly(A) tails of *ACTB* mRNAs were 3′ uridylated (Figures S4A–S4C), suggesting that mRNAs may be deadenylated prior to uridylation. Recent work indicates that mRNAs frequently have uridylate residues at the end of their poly(A) tail in nonapoptotic cells (Chang et al., 2014). It may be that full-length mRNAs are uridylated and then rapidly degraded by DIS3L2. Alternatively, another 3′ to 5′ exoribonuclease or endonuclease might digest deadenylated or polyadenylated mRNAs to initiate the decay pathway. Other candidate nucleases are the exosome, ERI1, and deadenylases (Hoefig et al., 2013; Schoenberg and Maquat, 2012). Knockdown of deadenylases and the exosome subunit *EXOSC10* yielded inconsistent results in preliminary studies. Their potential role in initiating apoptotic mRNA decay merits further investigation. Knockdown of the ER stress-induced endonuclease *IRE1* (Hollien and Weissman, 2006), unlike knock-down of *ZCCHC6*, *ZCCHC11*, and *DIS3L2*, promoted cell death (data not shown), making it unlikely that this nuclease is responsible for apoptotic mRNA decay.

We previously found that mRNA splicing and RNA export were disabled during both classical apoptosis and caspase-independent programmed cell death (Rajani et al., 2012). The resulting impaired translation of mRNAs induced in response to death stimuli promoted apoptosis by interfering with new protein synthesis needed for cellular repair. Now we find that preexisting mRNAs are rapidly degraded during classical apoptosis. Although we observed a stark reduction in poly(A) mRNAs during apoptosis, it is possible that some mRNAs are resistant to decay because of their subcellular localization or other features (such as structure). Deep sequencing of cellular RNAs in cells undergoing apoptosis could help to further delineate the apoptotic mRNA decay pathway.

Our results indicate that global apoptotic mRNA degradation arrests new protein synthesis, which is restored by knocking down *DIS3L2* (Figures 7J and 7K). mRNA decay occurs before alterations to translation initiation factors that have been previously implicated in translational arrest in nonapoptotic stress and apoptosis (Figures 3A and 3B). Since blocking mRNA decay with *DIS3L2* knockdown also inhibits apoptotic translation arrest, it is likely that mRNA decay is responsible for most, if not all, of the translation arrest that occurs during cell death.

How does mRNA decay promote cell death? Cell viability under basal and stressed conditions requires mRNA expression, as we confirmed in this study (Figure 4). In some settings, global mRNA decay might enhance cell death by reducing specific transcripts and their protein products. Reduced MCL-1, a protein that is rapidly degraded by the proteasome and by caspases during apoptosis, was largely responsible for the contribution of mRNA decay to the enhanced death of HeLa cells treated with STS. Increased MCL-1 with *DIS3L2* knockdown could also explain why MOMP and caspase 3 activation were reduced. Apoptotic signaling is amplified in a feedforward loop, as cyto-chrome *c* release leads to caspase activation, which triggers MOMP (Arnoult et al., 2003; Lakhani et al., 2006). Feedforward amplification is an important component of programmed cell death. Cells loaded with exogenous cytochrome *c* do not execute apoptosis without release of endogenous cytochrome *c* and caspase activation (Gabriel et al., 2003). This amplification could be attenuated by the persistence of MCL-1 in dying cells. However, MCL-1 is not expressed in all cells, and only some cells are sensitive to MCL-1 depletion (Petrocca et al., 2013). Therefore, decay of the *BCL2L3* transcript probably does not play an important role in promoting death in some cell types, where the loss of other mRNAs may be more critical. It is also possible that mRNA might bind and inhibit apoptotic proteins, and mRNA decay would relieve this inhibition. However, in one study (Mei et al., 2010), tRNA (but not mRNA) inhibited apoptosome formation and caspase activation, and in another study (Thomas et al., 2014), RNase treatment of cell lysates did not affect caspase 3 cleavage of some substrates. Nevertheless, mRNAs may influence caspase activation or specific activity in unknown ways. Further investigation of how mRNA decay promotes apoptosis in different settings is needed.

## EXPERIMENTAL PROCEDURES

### Cells

HeLa, Jurkat, HCT116, 721.221, and YT-Indy cells were obtained from ATCC. Jurkat, YT-Indy, and 721.221 cells were grown in RPMI with 10% heat-inactivated fetal bovine serum, 100 U/ml penicillin G, 100 μg/ml streptomycin sulfate, 6 mM HEPES, 1.6 mM L-glutamine, and 50 μM β-mercaptoethanol. Adherent cells were grown in DMEM with the same supplement. Stable HeLa-puro and HeLa-BCL2 cells were generated by retroviral infection and selection with puromycin using the pBABE-puro vector as described previously (Rajani et al., 2012).

### FISH

Adherent cells were grown in 24-well plates (Corning 3524) on 12-mm glass coverslips (VWR 89015-724) pre-treated with poly-L-lysine (Sigma P8920) for 5 min. Jurkat cells were washed, resuspended in PBS (Life Technologies 14190-144) with 0.5% BSA (Sigma A9647) and 2 mM EDTA (Life Technologies AM9260G), and then cytospun (Shandon Cytospin 3) for 4 min onto coverslips at 400 rpm before fixation. YT-Indy:721.221 immune conjugates were fixed first and then cytospun as described above. Cells were fixed for 10 min in 2% formaldehyde (Polysciences 18814) in 1× PBS and then permeabilized in pure methanol (Fisher BP1105-4) on dry ice for 10 min. Slides were washed three times for 5 min in 2× SSC. A control for each experiment was treated for 30 min in 0.1 M NaOH (Fisher SS255-1) in 2× SSC to hydrolyze all RNA. All coverslips were inverted onto a drop of hybridization buffer (10% dextran sulfate, 35% formamide, 0.3 M NaCl, 30 mM sodium citrate, 20 mM DTT) containing 1 μM *18S* rRNA probe (Cy5-ACCAGACTTGCCCTCC) and 333 nM poly(A) (Cy3-dT_50_) probe. Samples were placed in a humidified chamber and denatured for 5 min at 65°C, followed by 30 min at 45° C and at least 90 min at 42°C. The samples were washed three times for 5 min in 23 SSC at 37°C and stained with DAPI (Sigma D9542) in 2× SSC. Finally, the slides were mounted using polyvinyl alcohol (Sigma P-8136) aqueous mounting medium. Cells were imaged using an Axiovert 200M microscope (Pan Apochromat, 1.4 NA; Carl Zeiss) at 63×. Images were analyzed with SlideBook 4.2 (Intelligent Imaging Innovations). For quantification, cells were automatically identified based on the rRNA signal and background intensity was subtracted. All images shown are representative of at least three independent experiments.

### cRACE

Total RNA (20 μg) was treated with the DNA-free kit (Life Technologies) per the manufacturer’s instructions. RNA was ethanol precipitated overnight and washed twice with 70% ethanol. RNA was resuspended in 11 μl of 1× TAP buffer (Epicenter T19050). Half of this suspension was treated with 2 U of TAP for 1 hr at 37°C, and the other half was treated the same way except that TAP was not added. The reactions were then treated overnight at room temperature in a 100 μl volume with 10 U of T4 RNA ligase (NEB M0204S) with 1 mM ATP in 1× RNA ligase buffer. RNA was ethanol precipitated, washed, and resuspended in 20 μl dH_2_O. Then 3 μl of this RNA was mixed with 1 μl of 10 mM deoxyribonucleotide triphosphates (dNTPs), 2 μl of the *ACTB* RT primer, and 7 μl of dH_2_O. This was heated to 65°C for 5 min and cooled to 25°C. Then 7 μl of a master mix containing 4 μl 5× SuperScript III buffer, 1 μl 100 mM DTT, 1 μl SuperScript III (Life Technologies 18080-093) and 1 μl RNaseOUT (Life Technologies 10777-019) was added. The reaction was incubated at 55°C for 60 min and 70° C for 15 min before it was stored at 4° C. Then 2 μl of this reaction was mixed with 1 μl of each PCR primer (10 μM), 6 μl dH_2_O, and 10 μl 2× Phusion polymerase mix (NEB M0531L), and cycled as follows: one cycle at 98° C for 30 s; 35 cycles at 98° C for 10 s, 60° C for 10 s, and 72° C for 10 s; and one cycle at 72° C for 3 min. PCR products were gel extracted and cloned using the Zero-Blunt PCR kit (Life Technologies K2750) according to the manufacturer’s instructions. The primers used are listed in Table S1. In the figures, line diagrams depict the mapped 5′ and 3′ ends; the sequence between the primers is inferred to be full-length *ACTB* mRNA.

### RT-PCR with Adenylated Adaptor Primer to Detect Uridylated Decay Products

Total RNA (1 μg) was mixed with 3 μl of a 100 μM adaptor primer and 1 μl 10 mM dNTPs in a final volume of 13 μl. This was heated to 65° C for 5 min and cooled to 25° C. Then 7 μl of a master mix containing 4 μl 5× SuperScript III buffer, 1 μl 100 mM DTT, 1 μl SuperScript III, and 1 μl RNaseOUT was added. The reaction was incubated at 25° C for 10 min, 50° C for 30 min, and 70° C for 15 min before it was stored at 4° C. Then 2 μl of this reaction was mixed with 1 μl of each outer primer, 6 μl dH_2_O, and 10 μl 2× Phusion polymerase mix, and cycled as follows: one cycle at 98° C for 30 s; 30 cycles at 98° C for 10 s, 60° C for 10 s, 72° C for 10 s; and one cycle at 72° C for 3 min. PCR was repeated using identical parameters with the inner primers. Products were cloned as above. The primers used are listed in Table S1.

### Plasmids

GFP reporter constructs were kindly provided by B. Glaunsinger (Abernathy et al., 2014; Gaglia et al., 2012; Lee and Glaunsinger, 2009). GFP-DIS3L2 constructs were kindly provided by A. Dziembowski and M. Lubas (Lubas et al., 2013).

### Statistical Analysis

Pooled data from three independent cytotoxic attack FISH experiments were compared using a Mann-Whitney test. The cRACE results obtained after *DIS3L2* knockdown were compared by Fisher’s exact test. All other p values were computed using a two-tailed t test.

See Supplemental Experimental Procedures for additional methods.

## Supplementary Material

01

## Highlights

- Apoptosis triggers early global mRNA decay
- mRNA decay is triggered by mitochondrial outer membrane permeabilization
- mRNA decay products are 3′ uridylated by TUTases and degraded by DIS3L2
- DIS3L2 promotes apoptotic mRNA decay, translation arrest, and apoptosis

## References

[R1] Abernathy E, Clyde K, Yeasmin R, Krug LT, Burlingame A, Coscoy L, Glaunsinger B (2014). Gammaherpesviral gene expression and virion composition are broadly controlled by accelerated mRNA degradation. PLoS Pathog.

[R2] Aldridge BB, Gaudet S, Lauffenburger DA, Sorger PK (2011). Lyapunov exponents and phase diagrams reveal multi-factorial control over TRAIL-induced apoptosis. Mol. Syst. Biol.

[R3] Arnoult D, Gaume B, Karbowski M, Sharpe JC, Cecconi F, Youle RJ (2003). Mitochondrial release of AIF and EndoG requires caspase activation downstream of Bax/Bak-mediated permeabilization. EMBO J.

[R4] Bushell M, Stoneley M, Sarnow P, Willis AE (2004). Translation inhibition during the induction of apoptosis: RNA or protein degradation?. Biochem. Soc. Trans.

[R5] Chang H-M, Triboulet R, Thornton JE, Gregory RI (2013). A role for the Perlman syndrome exonuclease Dis3l2 in the Lin28-let-7 pathway. Nature.

[R6] Chang H, Lim J, Ha M, Kim VN (2014). TAIL-seq: genome-wide determination of poly(A) tail length and 3′ end modifications. Mol. Cell.

[R7] Degen WG, Pruijn GJ, Raats JM, van Venrooij WJ (2000). Caspase-dependent cleavage of nucleic acids. Cell Death Differ.

[R8] Del Prete MJ, Robles MS, Guáo A, Martĺnez-A C, Izquierdo M, Garcia-Sanz JA (2002). Degradation of cellular mRNA is a general early apoptosis-induced event. FASEB J.

[R9] Fan J, Yang X, Wang W, Wood WH, Becker KG, Gorospe M (2002). Global analysis of stress-regulated mRNA turnover by using cDNA arrays. Proc. Natl. Acad. Sci. USA.

[R10] Gabriel B, Sureau F, Casselyn M, Teissié J, Petit PX (2003). Retroactive pathway involving mitochondria in electroloaded cytochrome c-induced apoptosis. Protective properties of Bcl-2 and Bcl-XL. Exp. Cell Res.

[R11] Gaglia MM, Covarrubias S, Wong W, Glaunsinger BA (2012). A common strategy for host RNA degradation by divergent viruses. J. Virol.

[R12] Hoefig KP, Rath N, Heinz GA, Wolf C, Dameris J, Schepers A, Kremmer E, Ansel KM, Heissmeyer V (2013). Eri1 degrades the stem-loop of oligouridylated histone mRNAs to induce replication-dependent decay. Nat. Struct. Mol. Biol.

[R13] Holcik M, Sonenberg N (2005). Translational control in stress and apoptosis. Nat. Rev. Mol. Cell Biol.

[R14] Holler N, Zaru R, Micheau O, Thome M, Attinger A, Valitutti S, Bodmer J-L, Schneider P, Seed B, Tschopp J (2000). Fas triggers an alternative, caspase-8-independent cell death pathway using the kinase RIP as effector molecule. Nat. Immunol.

[R15] Hollien J, Weissman JS (2006). Decay of endoplasmic reticulum-localized mRNAs during the unfolded protein response. Science.

[R16] Hu W, Sweet TJ, Chamnongpol S, Baker KE, Coller J (2009). Co-translational mRNA decay in Saccharomyces cerevisiae. Nature.

[R17] Hu W, Petzold C, Coller J, Baker KE (2010). Nonsense-mediated mRNA decapping occurs on polyribosomes in Saccharomyces cerevisiae. Nat. Struct. Mol. Biol.

[R18] Jeffrey IW, Bushell M, Tilleray VJ, Morley S, Clemens MJ (2002). Inhibition of protein synthesis in apoptosis: differential requirements by the tumor necrosis factor α family and a DNA-damaging agent for caspases and the double-stranded RNA-dependent protein kinase. Cancer Res.

[R19] Lakhani SA, Masud A, Kuida K, Porter GA, Booth CJ, Mehal WZ, Inayat I, Flavell RA (2006). Caspases 3 and 7: key mediators of mitochondrial events of apoptosis. Science.

[R20] Lee YJ, Glaunsinger BA (2009). Aberrant herpesvirus-induced polyadenylation correlates with cellular messenger RNA destruction. PLoS Biol.

[R21] Lubas M, Damgaard CK, Tomecki R, Cysewski D, Jensen TH, Dziembowski A (2013). Exonuclease hDIS3L2 specifies an exosome-independent 3′-5′ degradation pathway of human cytoplasmic mRNA. EMBO J.

[R22] Malecki M, Viegas SC, Carneiro T, Golik P, Dressaire C, Ferreira MG, Arraiano CM (2013). The exoribonuclease Dis3L2 defines a novel eukaryotic RNA degradation pathway. EMBO J.

[R23] Mei Y, Yong J, Liu H, Shi Y, Meinkoth J, Dreyfuss G, Yang X (2010). tRNA binds to cytochrome c and inhibits caspase activation. Mol. Cell.

[R24] Morley SJ, Coldwell MJ, Clemens MJ (2005). Initiation factor modifications in the preapoptotic phase. Cell Death Differ.

[R25] Mullen TE, Marzluff WF (2008). Degradation of histone mRNA requires oligouridylation followed by decapping and simultaneous degradation of the mRNA both 5′ to 3′ and 3′ to 5′. Genes Dev.

[R26] Nijhawan D, Fang M, Traer E, Zhong Q, Gao W, Du F, Wang X (2003). Elimination of Mcl-1 is required for the initiation of apoptosis following ultraviolet irradiation. Genes Dev.

[R27] Norbury CJ (2013). Cytoplasmic RNA: a case of the tail wagging the dog. Nat. Rev. Mol. Cell Biol.

[R28] Orlando KA, Stone NL, Pittman RN (2006). Rho kinase regulates fragmentation and phagocytosis of apoptotic cells. Exp. Cell Res.

[R29] Petrocca F, Altschuler G, Tan SM, Mendillo ML, Yan H, Jerry DJ, Kung AL, Hide W, Ince TA, Lieberman J (2013). A genome-wide siRNA screen identifies proteasome addiction as a vulnerability of basal-like triple-negative breast cancer cells. Cancer Cell.

[R30] Rajani DK, Walch M, Martinvalet D, Thomas MP, Lieberman J (2012). Alterations in RNA processing during immune-mediated programmed cell death. Proc. Natl. Acad. Sci. USA.

[R31] Riedl SJ, Shi Y (2004). Molecular mechanisms of caspase regulation during apoptosis. Nat. Rev. Mol. Cell Biol.

[R32] Rissland OS, Norbury CJ (2009). Decapping is preceded by 3′ uridylation in a novel pathway of bulk mRNA turnover. Nat. Struct. Mol. Biol.

[R33] Saelens X, Kalai M, Vandenabeele P (2001). Translation inhibition in apoptosis: caspase-dependent PKR activation and eIF2-alpha phosphorylation. J. Biol. Chem.

[R34] Schmidt M-J, West S, Norbury CJ (2011). The human cytoplasmic RNA terminal U-transferase ZCCHC11 targets histone mRNAs for degradation. RNA.

[R35] Schoenberg DR, Maquat LE (2012). Regulation of cytoplasmic mRNA decay. Nat. Rev. Genet.

[R36] Shen B, Goodman HM (2004). Uridine addition after microRNA-directed cleavage. Science.

[R37] Slevin MK, Meaux S, Welch JD, Bigler R, Miliani de Marval PL, Su W, Rhoads RE, Prins JF, Marzluff WF (2014). Deep sequencing shows multiple oligouridylations are required for 3′ to 5′ degradation of histone mRNAs on polyribosomes. Mol. Cell.

[R38] Tani H, Mizutani R, Salam KA, Tano K, Ijiri K, Wakamatsu A, Isogai T, Suzuki Y, Akimitsu N (2012). Genome-wide determination of RNA stability reveals hundreds of short-lived noncoding transcripts in mammals. Genome Res.

[R39] Taylor RC, Cullen SP, Martin SJ (2008). Apoptosis: controlled demolition at the cellular level. Nat. Rev. Mol. Cell Biol.

[R40] Thomas MP, Lieberman J (2013). Live or let die: posttranscriptional gene regulation in cell stress and cell death. Immunol. Rev.

[R41] Thomas MP, Whangbo J, McCrossan G, Deutsch AJ, Martinod K, Walch M, Lieberman J (2014). Leukocyte protease binding to nucleic acids promotes nuclear localization and cleavage of nucleic acid binding proteins. J. Immunol.

[R42] Thornton JE, Chang H-M, Piskounova E, Gregory RI (2012). Lin28-mediated control of let-7 microRNA expression by alternative TUTases Zcchc11 (TUT4) and Zcchc6 (TUT7). RNA.

[R43] Thornton JE, Du P, Jing L, Sjekloca L, Lin S, Grossi E, Sliz P, Zon LI, Gregory RI (2014). Selective microRNA uridylation by Zcchc6 (TUT7) and Zcchc11 (TUT4). Nucleic Acids Res.

[R44] Ustianenko D, Hrossova D, Potesil D, Chalupnikova K, Hrazdilova K, Pachernik J, Cetkovska K, Uldrijan S, Zdrahal Z, Vanacova S (2013). Mammalian DIS3L2 exoribonuclease targets the uridylated precursors of let-7 miRNAs. RNA.

[R45] Weng C, Li Y, Xu D, Shi Y, Tang H (2005). Specific cleavage of Mcl-1 by caspase-3 in tumor necrosis factor-related apoptosis-inducing ligand (TRAIL)-induced apoptosis in Jurkat leukemia T cells. J. Biol. Chem.

