# Supplementary material for: Apoptosis Triggers Specific, Rapid, and Global mRNA Decay with 3′ Uridylated Intermediates Degraded by DIS3L2
Source: Cell Rep. Author manuscript; Available in PMC 2016 May 10. (PMC4862650; doi:10.1016/j.celrep.2015.04.026)
Supplement: 01 [file NIHMS761190-supplement-01.pdf]

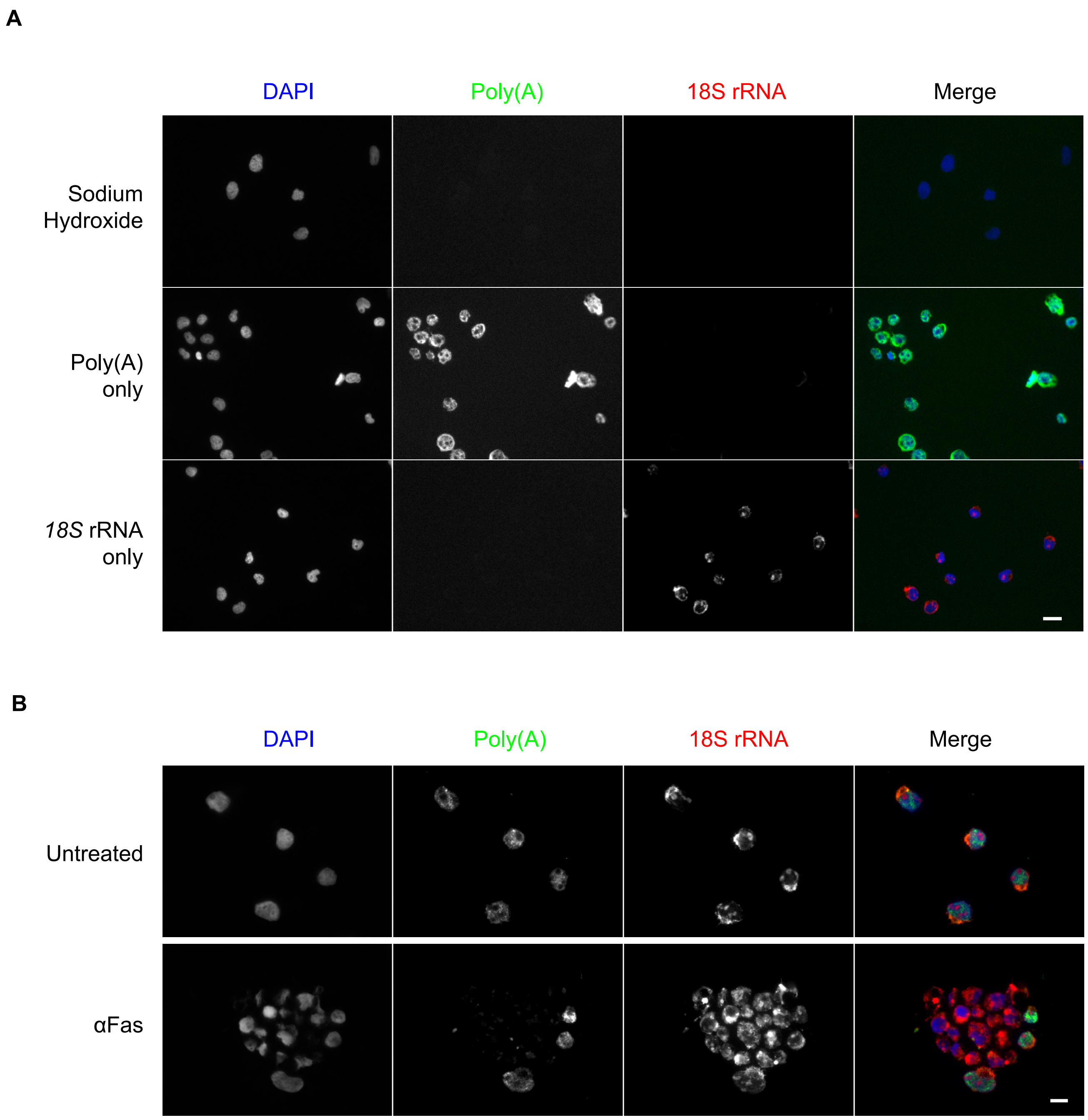

Figure S1
Validation of RNA FISH to measure mRNA decay, related to Figure 1.
Jurkat cells were cytospun onto coverslips and stained by RNA FISH according to the experimental methods.
(A) FISH performed on untreated Jurkat cells confirmed the specificity of poly(A) and *18S* FISH. Sodium hydroxide treatment, which hydrolyzes RNA, but not DNA, abolished mRNA and rRNA staining, but not DAPI staining. This strongly suggests that the FISH was specific for RNA.
(B) Jurkat cells were treated with αFas for 3 hr in the presence of blebbistatin, an inhibitor of blebbing. Apoptosis was confirmed by annexin V staining (data not shown). mRNA still disappeared in non-blebbing αFas-treated cells undergoing apoptosis. The minority of cells with persistent poly(A) signal had DAPI staining that showed non-apoptotic nuclei.
Scale bar represents 10 μm.

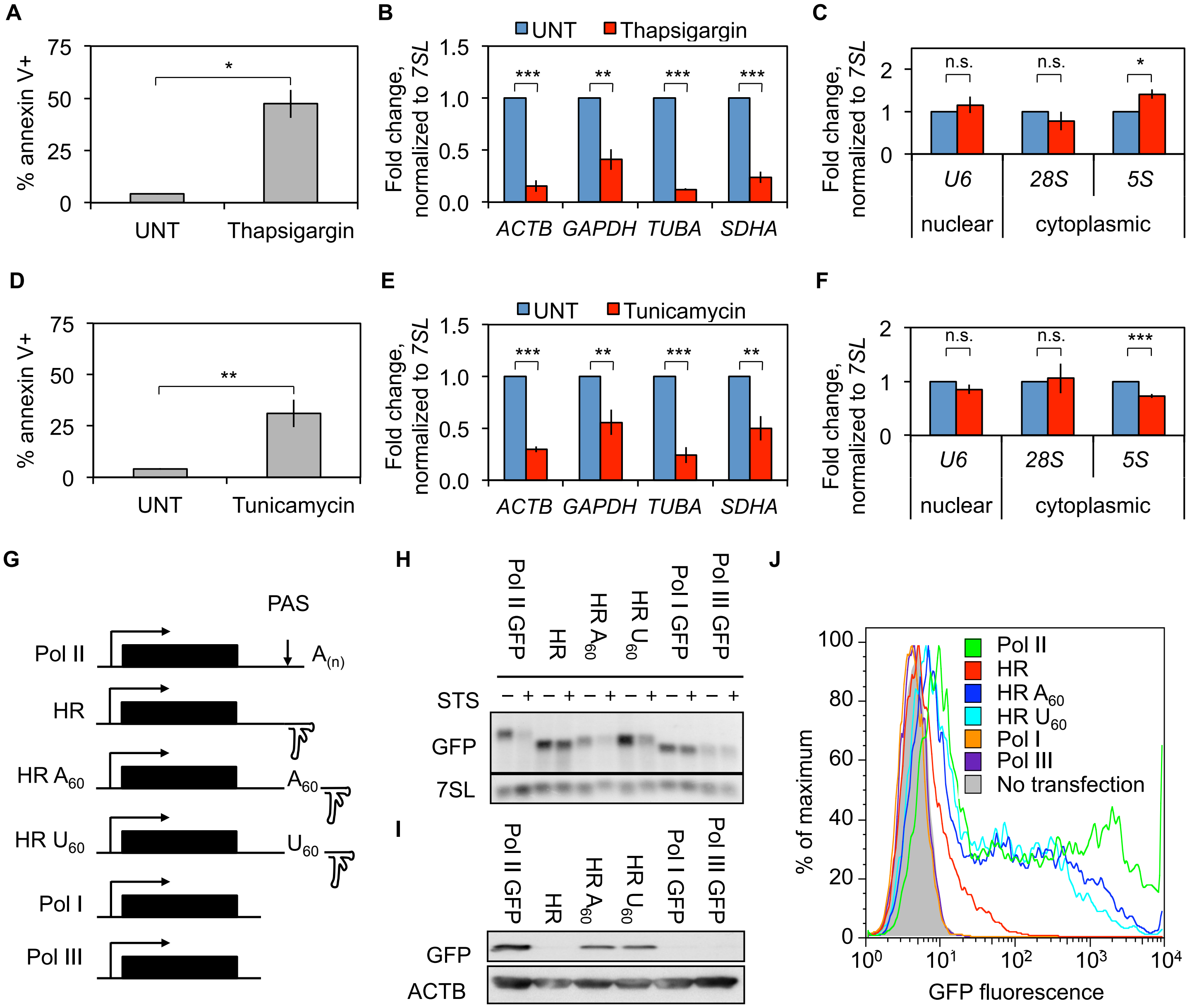

Figure S2

Apoptotic RNA decay occurs in many cell types responding to diverse stimuli, and is specific to mRNAs. Related to Figure 2.

(A-C) HCT116 cells were treated with thapsigargin, or left untreated, for 12 hrs, then cells were harvested for annexin V staining (A) or RNA was harvested and the indicated mRNAs (B) or ncRNAs (C) were assayed by qRT-PCR. mRNAs consistently declined, while ncRNAs were more stable.

(D-F) HCT116 cells were treated with tunicamycin, or left untreated, for 12 hrs, then cells were harvested for annexin V staining (D) or RNA was harvested and the indicated mRNAs (E) or ncRNAs (F) were assayed by qRT-PCR. mRNAs consistently declined, while ncRNAs were more stable.

(G-J) The indicated reporters (G) were transfected into HeLa cells, 48 hr later the cells were treated with STS or left untreated. GFP RNA expression was analyzed by Northern blot (H). The Northern blot probe targeted the GFP coding region. All of the constructs produced GFP RNA when introduced into cells. GFP protein expression was assayed by immunoblot (I) and flow cytometry (J). There was high protein expression from transcripts driven by a polymerase II (Pol II) promoter. When the polyadenylation signal sequence (PAS) was replaced by a self-cleaving hammerhead ribozyme (HR), protein expression was lost, but rescued by the addition of 60 adenine or 60 uridine nucleotides before the HR sequence. Pol I and Pol III transcripts expressed no protein. Although all of the RNAs were expressed, only translated species decayed (H).

Error bars represent SEM of at least three independent experiments. * $p<0.05$; ** $p<0.01$; *** $p<0.001$.

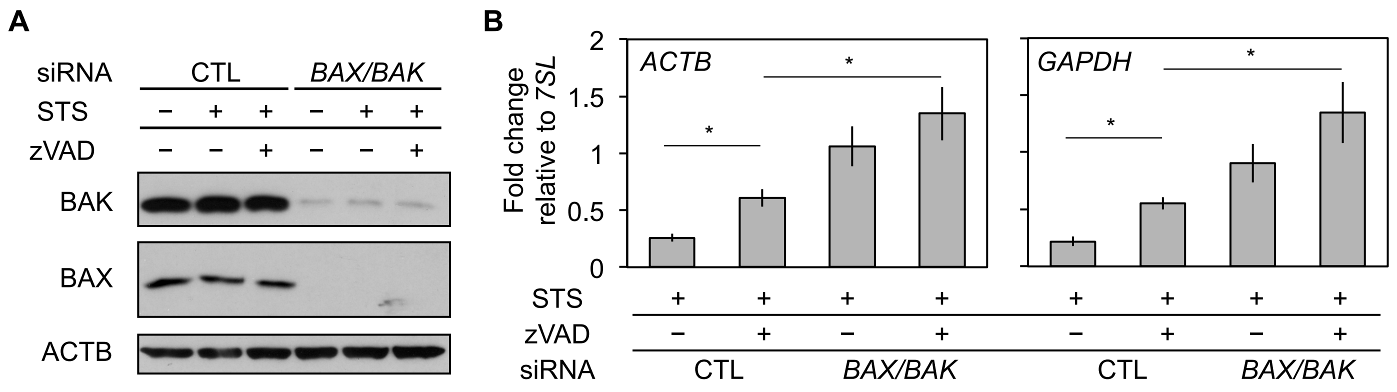

Figure S3

Apoptotic mRNA decay is dependent on the mitochondrial proteins BAX and BAK. Related to Figure 3.

HeLa cells were transfected with a control siRNA (CTL) or pooled siRNAs targeting *BAX* and *BAK*. 72 hr later, the cells were treated with STS±zVAD and harvested for immunoblot of the indicated proteins (A) or qRT-PCR for the indicated RNAs. Paired *BAX/BAK* knockdown rescued mRNA levels to a greater extent than zVAD, suggesting that mRNA decay depends of full activation of the mitochondrial apoptotic pathway. Error bars represent SEM of at least 3 independent experiments. * $p<0.05$.

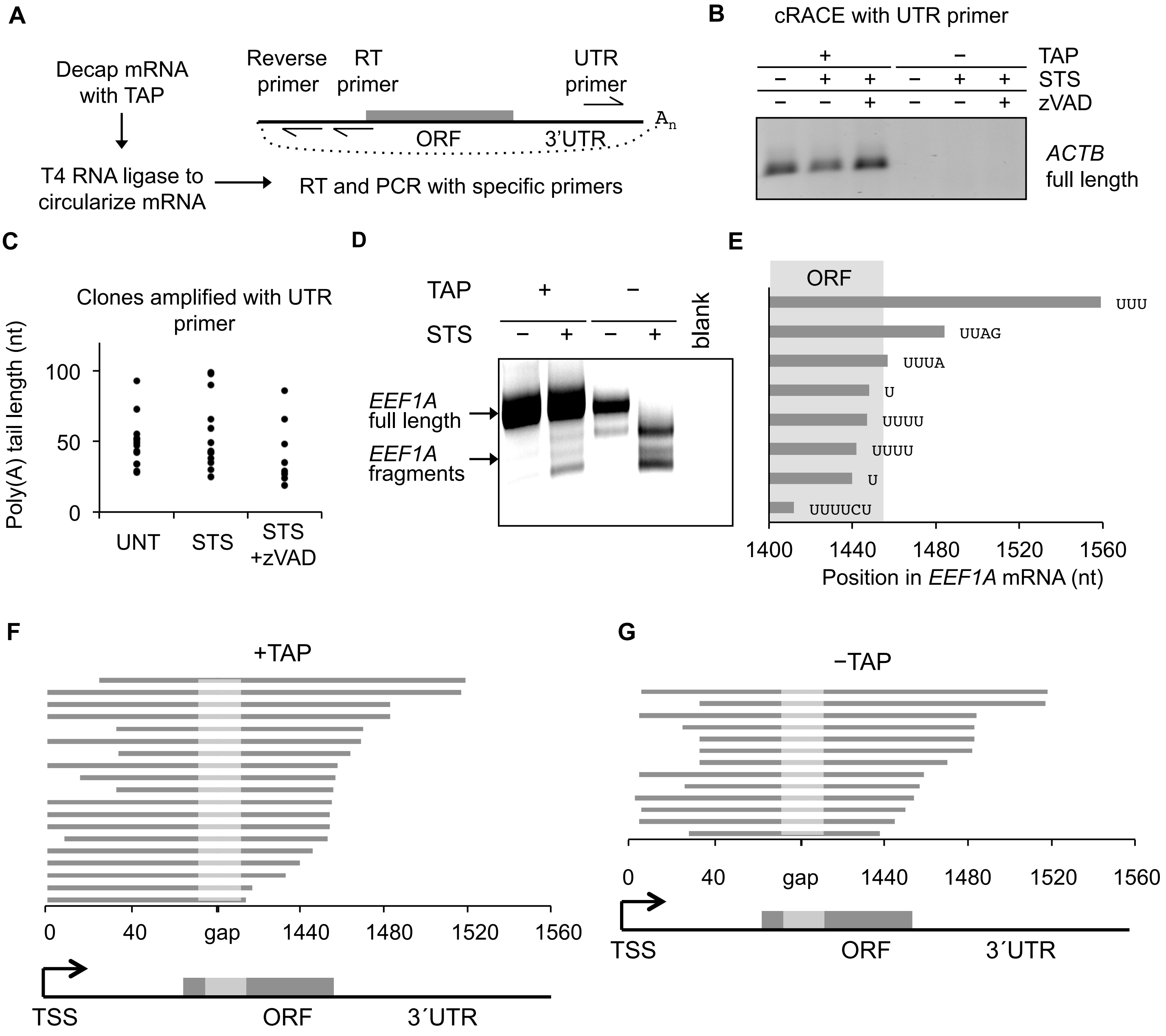

Figure S4

Characterization of mRNA decay products in cell death, related to Figure 5.

(A) cRACE was performed on *ACTB* mRNA with a forward primer targeting the 3´UTR.

(B) Gel electrophoresis of amplified *ACTB* junctions indicates that the full-length *ACTB* mRNA remained mostly capped, even in apoptosis.

(C) cRACE clones were sequenced and poly(A) tail lengths of capped, polyadenylated *ACTB* mRNA termini amplified with a primer targeting the *ACTB* 3´UTR are plotted. Poly(A) lengths did not change significantly with treatment. Clones were all isolated in one experiment.

(D-G) *EEF1A* mRNAs were amplified by cRACE with a forward primer in the ORF. PCR products were run on a gel (D) and the fragments were isolated by gel purification. Some *EEF1A* decay products with intact 5´ ends had nontemplated tails, which were rich in uridylates (E). As with the *ACTB* mRNA decay products, most clones derived from TAP-treated RNA had intact 5´ ends (F), while all clones from TAP-untreated RNA had evidence of 5´ to 3´ decay (G). Clones in E-G were all isolated in one experiment.

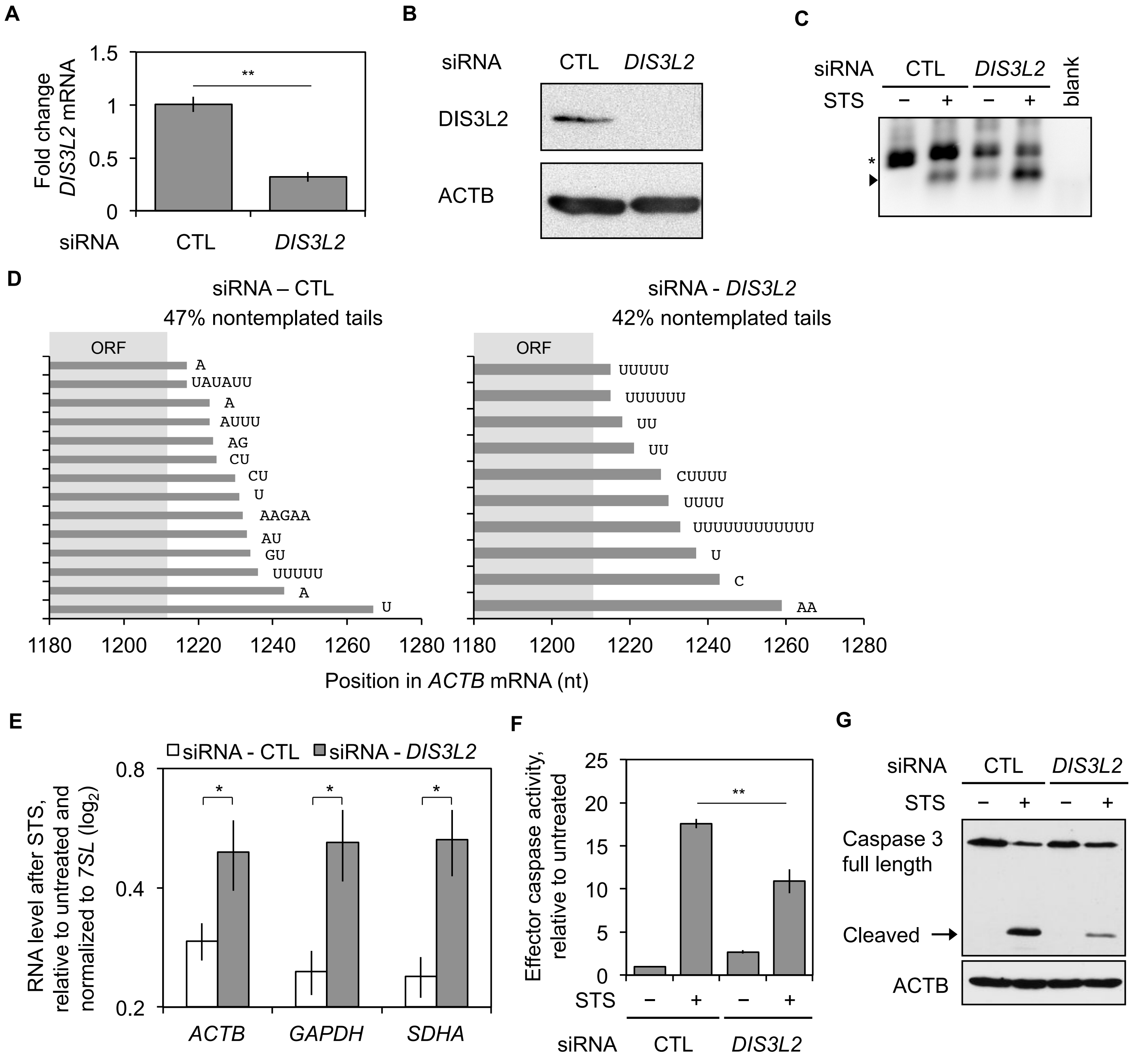

Figure S5

DIS3L2 degrades U-tailed decay intermediates during cell death, related to Figure 7.

(A,B) qRT-PCR (A) and immunoblot (B) confirmed siRNA knockdown of *DIS3L2* in HeLa cells. A different siRNA sequence was used (A-G) than in the main text (see methods).

(C,D) HeLa cells were transfected with CTL or *DIS3L2* siRNAs, then treated with STS for 4 hr. Total RNA was harvested and subjected to U-tailing assays. U-tailed *ACTB* decay intermediates (arrow) accumulated in STS-treated HeLa cells after *DIS3L2* knockdown as measured by RT-PCR (C) and cRACE (D). U-tailed intermediates also accumulated in untreated living cells after *DIS3L2* knockdown (C). A summary of the cRACE results with statistical analysis is presented in Figure 7C.

(E-G) *DIS3L2* knockdown in HeLa cells partially restored mRNA levels (E) and reduced effector caspase activation (F) and caspase 3 cleavage (G) following 4 hr of treatment with STS.

Error bars represent SEM of at least 3 independent experiments. * $p<0.05$; ** $p<0.01$.

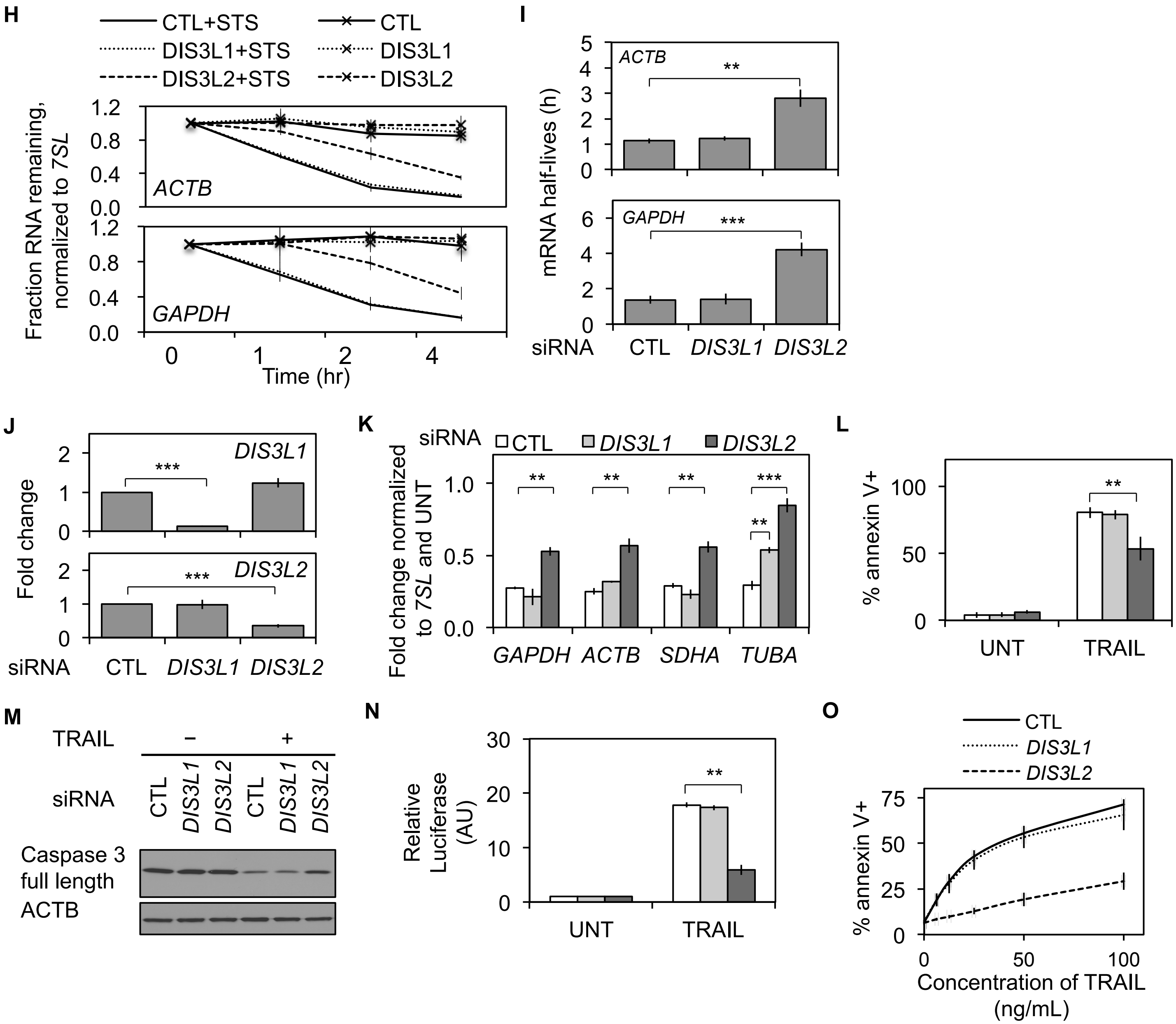

Figure S5 (continued)

DIS3L2 degrades U-tailed decay intermediates during cell death, related to Figure 7.

(H,I) HeLa cells were transfected with the indicated siRNA pools and RNA was analyzed by qRT-PCR relative to *7SL* at the indicated times after adding α-amanitin+/-STS (H). The mRNA half-lives in STS-treated Hela cells were determined using the one-phase exponential decay equation (I). *DIS3L2*, but not *DIS3L1*, siRNAs increased mRNA half-life after STS treatment.

(J-N). HCT116 cells were transfected with CTL, *DIS3L1* or *DIS3L2* siRNAs, then treated with TRAIL or left untreated for 3 hrs. Knockdown was confirmed by qRT-PCR (J), mRNA levels were measured by qRT-PCR relative to *7SL* (K), apoptosis was assayed by annexin V staining (L), and caspase activation was measured by immunoblot (M) and a luminescent assay (N). *DIS3L2*, but not *DIS3L1* siRNAs rescued mRNA levels, apoptosis, and caspase 3 cleavage and activation.

(O) HeLa cells were transfected with the indicated siRNA pools, then treated with the indicated dose of TRAIL and stained with annexin V. *DIS3L2*, but not *DIS3L1*, siRNAs reduced apoptosis in response to all of the different doses of TRAIL.

Error bars represent SEM of at least 3 independent experiments. * $p<0.05$; ** $p<0.01$; *** $p<0.001$.

Cell treatments Jurkat cells were treated with 1:1000 αFas antibody (Millipore, clone CH11). All cells except HeLa were treated with 2 μg/mL ActD (Sigma A9415) to inhibit transcription. HeLa cells were treated with 10 μg/mL ActD (Fig. 1E), 100 ng/mL superkiller TRAIL (Enzo Life Sciences ALX-201-115-C010), 20 μg/mL α-amanitin (Sigma, A2263-1MG), 500 nM STS (Cell Signaling #9953), 50 μg/mL tunicamycin (Sigma, T7765-10MG), or 500 μM etoposide (Sigma, E1383-25MG) unless otherwise indicated. HCT116 cells were treated with 100 ng/mL superkiller TRAIL, 1 μg/mL tunicamycin, or 1 μM thapsigargin (Sigma, T9033-.5MG). To inhibit caspase activity, cells were treated with 100 μM zVAD (BD 550377). ActD and zVAD were dissolved in DMSO; an equivalent amount of DMSO (Sigma D2650) was added to all control treatments to equalize the final DMSO concentration. For cytotoxic attack experiments, YT-Indy cells were mixed with 721.221 cells at an effector:target ratio of 3:1, which led to 60% apoptosis of the target 721.221 cells (Rajani et al., 2012) .

Assessment of apoptosis For cytotoxic attack experiments, death was verified by ^{51}Cr release assays as previously described (Rajani et al., 2012). In other experiments, cells were stained for annexin V-APC (Life Technologies A35110) or $DiIC_1(5)$ (Life Technologies M34151) according to the manufacturer's instructions. Both dyes were measured on a FACSCalibur flow cytometer. For measurements of effector caspase activity and cell survival, cells, plated on 96 well tissue culture plates (Corning 3610) one day before treatment, were treated for the indicated time, and activity was assessed with the Caspase-Glo (Promega G8090) or CytoTox-Glo (Promega G9290) kits, respectively, according to the manufacturer's instructions. Luminescence was measured on a BioTek Synergy 2 plate reader. For cell fractionation, cells were washed with PBS and harvested

by scraping in PBS on ice. Cells were washed once in PBS and resuspended in 5 cell volumes of mitochondrial isolation buffer (250 mM sucrose, 10 mM Tris-HCl (pH 7.0), 100 mM KCl, 1 mM EDTA, 1X protease inhibitors (Roche 04 693 159 001)). Cells were incubated for 30 min in this buffer on ice, then lysed by passage through a 27 gauge needle 30 times. Lysates were spun at 800 x g for 10 min to remove cell debris. The supernatant was spun again at maximum speed for 25 min and this supernatant (cytosolic fraction) was suspended in RIPA buffer for immunoblotting. Total protein was harvested in RIPA buffer to control for non-cytosolic proteins. For DNA extraction, cells were resuspended and vortexed in lysis buffer (10 mM Tris-HCl, pH 8.0, 10 mM EDTA pH 8.0, 100 mM NaCl, 0.5% SDS with 40 μg/mL proteinase K (Sigma)) and incubated for 3 hr at 56°C. Lysate was mixed with an equal volume of TE-buffered phenol, pH 8.0 (Sigma P4557) and vortexed well before centrifugation for 10 min at 13,000 rpm. The aqueous phase was extracted once with an equal volume of chloroform, ethanol precipitated, and dissolved in TE. DNA laddering was analyzed by agarose gel electrophoresis.

Clonogenic survival assay Cells transfected with the indicated siRNAs 48 hr earlier were replated on 6 well plates at a density of 300 cells/well. 24 hr after replating, cells were treated with 50 ng/μl TRAIL or STS (250 nM or 500 nM) for 2 hr. The cells were washed once with PBS and fresh medium was added. The colonies were grown until each colony reached ~50 cells (~9 days). Cells were fixed in 10% formalin for 5 min, washed once in PBS, and then stained for 15 min with crystal violet (20% methanol, 0.1% crystal violet). Cells were then rinsed in water; colonies were counted manually.

qRT-PCR RNA was harvested and extracted with TRIzol reagent (Life Technologies 15596018) according to the manufacturer's instructions. 500-1000 ng of total RNA was first treated with the DNA-free kit (Life Technologies AM1906) according to the manufacturer's instructions, and half was reverse transcribed in a 20 µl volume with iScript RT reagent (BioRad 1708841) according to the manufacturer's instructions. The other half was used as a no RT control. qRT-PCR was performed with SsoFast reagent (BioRad 172-5204) in 20 µl reaction volumes with 1 µl of cDNA and 500 nM primers, using a BioRad CFX96 PCR machine with the recommended cycling parameters for the Ssofast reagent. Primers are given in Table S1. Taqman miRNA qRT-PCR was performed according to the manufacturer's instructions (Life Technologies N808-0234). The following kits from Life Technologies were used for reverse transcription and qPCR. miR-16: #000391, miR-21: #000397, U6: #001093.

Western blotting Western blotting was performed as described previously (Rajani et al., 2012). All primary antibodies were used at 1:1000 and secondary antibodies at 1:2500. The following antibodies were used: ACTB (DSHB, #JLA20-c); PARP1 (Santa Cruz, H-250); eIF4G (Cell Signaling, 2498); phospho-eiF2α (Cell Signaling, 9721); total eiF2α (Cell Signaling, 9722); VDAC1 (Santa Cruz, sc-8828); cytochrome c (Biolegend, 612504); Caspase 3 (Cell Signaling, 9662); DIS3L1 (Abcam, ab89042); DIS3L2 (Novus, NBP1-84740); GFP (ABM, G160); XIAP (Cell Signaling, 2045); BCL-x_L (Cell Signaling, 2764); Caspase 8 (Cell Signaling, 9746); MCL-1 (Cell Signaling, 4572); BCL2 (Cell Signaling, 2874); BID (Cell Signaling, 2006), Caspase 9 (Cell Signaling, 9502); BAX (Santa Cruz, N-20); BIM (Santa Cruz, H-191); Anti-Mouse HRP (GE Healthcare, NA931V); Anti-Rabbit HRP (GE Healthcare, NA934V); Anti-Chicken HRP

(Abcam, ab6753-1500); Anti-Goat HRP (Santa Cruz, sc-2020).

Northern blotting 2 µg of total RNA was ethanol-precipitated, washed, and dissolved in gel loading buffer (Life Technologies AM8547). RNA was denatured and run on a 1.5% denaturing agarose gel in MOPS buffer (Life Technologies AM8671). rRNA was visualized with ethidium bromide staining, and the RNA was transferred to a Nytran (GE Healthcare 10416296) membrane in SSC (Life Technologies AM9763). Probes were prepared by in vitro transcription (AM1314) of PCR templates. Samples were hybridized in Ultrahyb buffer (Life Technologies AM8670) and washed twice at 65°C for 5 min in 2X SSC, 0.1% SDS, then twice at 65°C for 15 min in 0.1X SSC, 0.1% SDS before autoradiography.

siRNA knockdown of target genes HeLa cells were plated on 6 well plates at a density of $8x10^4$ cells per well 1 day before transfection. For transfection, cells were washed twice with PBS and 867 µl of OptiMEM (Life Technologies 51985-091) was added to each well. 5 µl OligoFectamine (Life Technologies 12252-011) was mixed with 24 µl OptiMEM and allowed to rest for 5 min at RT. 100 µl OptiMEM pre-mixed with 2.5 µl of a 20 µM siRNA (or siRNA pool) was added to the OligoFectamine complexes, mixed well, and incubated for 20 min at RT. The entire reaction mixture was added to 1 well of cells. After 6 hr, the cells were washed in PBS and returned to growth medium. Unless otherwise indicated, all assays were performed 72 hr after transfection. *ZCCHC6/ZCCHC11* knockdown required double transfection. Briefly, HeLa cells were initially transfected with siRNAs in suspension using Lipofectamine RNAiMAX (Life Technologies 13778-075) according to the manufacturer's instructions. One day later, adherent cells were re-transfected with the same siRNAs.

siRNAs The following siRNA pools were from Dharmacon, used in Figure 7, Figure S5H,I and O, and Figure S3: CTL (D-001210-05-05); BAX (M-003308-03-0005); BAK (M-003305-02-0005); DIS3L1 (M-015333-00-0005); DIS3L2 (M-018715-01-0005); BCL2L3 (MCL-1) (M-004501-08-0005). In Figure 7C and Figure S5A-G, the following siRNAs were used (from Ambion): CTL (4390843); DIS3L2 (s43418). In Figure 6, the following siRNAs were used (from Ambion): CTL (4390843); ZCCHC6 (s36060); ZCCHC11 (s235511); PAPD7 (s21760). In Figure S5J-N, the following siRNAs were used (from Ambion): CTL (4390843); DIS3L1 (s41866); DIS3L2 (s43419).

Ambion siRNAs

Gene name	ID	Sequence
DIS3L1	s41866	GUUUGACCAUGUAACCGUAtt
DIS3L2	s43418	GCACCAAACUUAGCUACGAtt
DIS3L2	s43419	GCUGCGUAUGAAUCAGAUAtt
PAPD7	s21760	CCGGUAUUAGAAUCAAAGAtt
ZCCHC6	s36060	GGCUGGAAAUUAAACGUAUtt
ZCCHC11	s23551	GGAUUUGGAUUUCGUGAUAtt

Dharmacon siRNA pools

Gene name	ID	Sequence
BAK	M-003305-02-0005	GCUUCGUGGUCGACUUCAU
		CGACAUCAACCGACGCUAU
		CAACCGACGCUAUGACUCA
		CAGAGAAUGCCUAUGAGUA
BAX	M-003308-03-0005	CAUUGGACUUCCUCCGGGA
		ACAUGUUUUCUGACGGCAA
		CUGAGCAGAUCAUGAAGAC
		GAACUGAUCAGAACCAUCA
BCL2L3	M-004501-08-0005	CGAAGGAAGUAUCGAAUUU
		AGAACGAAUUGAUGUGUAA
		GGACCAACUACAAAUUAAU
		GCUACGUAGUUCGGGCAAA

Gene name	ID	Sequence
DIS3L1	M-015333-00-0005	GAUGGAAACUUAAGCGUUG
		CCACUUUACUUCUCCAAUA
		CGAAACCGCUCAAUUCAUG
		UCACGUUGCCAUUCUGAUA
DIS3L2	M-018715-01-0005	CAGCAAAGGUGGUUUACAU
		UGGCACGGCCUAAAGAUUA
		CGUAAUAGAGCCUUAAAUG
		CCGAGUGCCUAGAAUUUAU

Cloning The *BCL2* expression vector was generated by PCR amplification of *BCL2* and subcloning into the EcoRI and SalI sites of pBABE-puro. Multiple clones were picked and correct insertions/mutations were verified by sequencing. Primer sequences are provided in Table S1.

^{35}S incorporation assay Cells were grown in methionine- and cysteine-free medium supplemented with 5% dialyzed serum for 30 min, before the medium was replaced with methionine- and cysteine-free DMEM containing 40 μCi/ml ^{35}S-cysteine/methionine (EXPRE^{35}S^{35}S Protein Labeling Mix, PerkinElmer NEG072002MC) and 5% dialyzed serum. After 30 min of labeling, the cells were washed with phosphate-buffered saline and lysed with Laemmli sample buffer. Proteins were resolved by SDS-PAGE, transferred to a PVDF membrane, and detected by autoradiography.

qRT-PCR primers

Target	Forward	Reverse
28S rRNA	TCATCAGACCCCAGAAAAGG	GATTCGGCAGGTGAGTTGTT
5S rRNA	GGCCATACCACCCTGAACGC	CAGCACCCGGTATTCCCAGG
7SL	ATCGGGTGTCCGCACTAAGTT	CAGCACGGGAGTTTTGACCT
U6	CGCTTCGGCAGCACATATAC	CGAATTTGCGTGTCATCCTT
U2	TGGAGCAGGGAGATGGAATA	CGTTCCTGGAGGTACTGCAA
ACTB	AAGGCCAACCGCGAGAAGAT	ACAGCCTGGATAGCAACGTACA
GAPDH	AAGGTGAAGGTCGGAGTCAAC	GGGGTCATTGATGGCAACAATA
SDHA	TGGGAACAAGAGGGCATCTG	CCACCACTGCATCAAATTCATG
LMNB1	TGCTACTGCACTTGGTGACA	AGGCTCTGACAACGATTCTCCA
STAT5B	AAATTCAAGGCCGAAGTGCAGAGC	CATCACACCGTCAAACCATTGCCA
APAF1	GTCACCATACATGGAATGGCA	CTGATCCAACCGTGTGCAAA
TUBA	TCTGTGAAACTGGTGCTGGA	AGTGACCACGGGCATAGTTGTT
DIS3L1	CTTTTGTTCGACTTCAAGGAGCC	CCGTGGATTAGGATGTCACTGA
DIS3L2	ACCGCGAGAGCAACAAGCT	GATCTTGTGGGCCACTGC
ZCCHC6	ATAACACCAGGGAACTATGGGA	CATTCATCCAAGCGGGTTGAC
ZCCHC11	AAAAGGGACCCAGTTTACTGTTG	GTCCGATACGTCTTCAATTCCTG

Cloning primers

Plasmid name	Forward Primer	Reverse Primer
pBABE-BCL2	ATCGAATTCGGAAGGATGGCGCACGCTGGGAGA	ATCGTCGACTCACTTGTGGCCCAGATAGG

cRACE primers

Name	Sequence
ACTB RT primer	GCTGGCGGCGGGTGTG
ACTB Reverse primer	GCAAAGGCGAGGCTCTGTG
ACTB UTR Forward primer	CCAGTTGAATAAAAGTGCACACC
ACTB ORF Forward primer	TCCATCGTCCACCGCAAATG
EEF1A RT primer	CGACAATGTTGATATGAGTCTTTTC
EEF1A Reverse primer	TTAGGGGTAGTTTTCACGACAC
EEF1A Forward primer	TGTCATCAAAGCAGTGGACAAG

RT for uridylated products primers

RT Adapter	GCGAGCACAGAATTAATACGACTCACTATAGGAAAAAAAAAAAA
OUTER_REV	GCGAGCACAGAATTAATACGACT
OUTER_ACTB_FWD	AAGCAGGAGTATGACGAGTCC
INNER_REV	CGCGGATCCGAATTAATACGACTCACTATAGG
INNER_ACTB_FWD	TCCATCGTCCACCGCAAATG

Supplemental Table 1: Primers used in this study, related to Experimental Procedures
